# Strain profiling and epidemiology of bacterial species from metagenomic sequencing

**DOI:** 10.1038/s41467-017-02209-5

**Published:** 2017-12-22

**Authors:** Davide Albanese, Claudio Donati

**Affiliations:** Computational Biology Unit, Research and Innovation Centre, Fondazione Edmund Mach, Via Edmund Mach 1, 38010 San Michele all’Adige, Italy

## Abstract

Microbial communities are often composed by complex mixtures of multiple strains of the same species, characterized by a wide genomic and phenotypic variability. Computational methods able to identify, quantify and classify the different strains present in a sample are essential to fully exploit the potential of metagenomic sequencing in microbial ecology, with applications that range from the epidemiology of infectious diseases to the characterization of the dynamics of microbial colonization. Here we present a computational approach that uses the available genomic data to reconstruct complex strain profiles from metagenomic sequencing, quantifying the abundances of the different strains and cataloging them according to the population structure of the species. We validate the method on synthetic data sets and apply it to the characterization of the strain distribution of several important bacterial species in real samples, showing how its application provides novel insights on the structure and complexity of the microbiota.

## Abstract (web summary)

Microbiota is often a complex mixture of multiple coexisting species and strains with high level of phenotypic and genomic variability. Here, Albanese and Donati develop StrainEst for estimating the number and identity of coexisting strains and their relative abundances in mixed metagenomic samples.

## Introduction

Next-generation sequencing technologies provide for the first time the possibility to study the composition of complex microbial communities in human-derived and environmental samples in a culture-independent manner. Thanks to the large amount of data provided by these techniques, it is now widely accepted that the microbiota plays a role in maintaining host health^1,2^, and that alterations of the equilibrium between host and colonizing microbiota is implicated in a number of diseases^3^. However, to fully exploit the potential of metagenomics in clinical and epidemiological applications, computational techniques able to profile microbial communities at resolution beyond the species level are needed, given the high level of phenotypic and genomic variability between strains of the same species^4^.

Widely used marker-based computational methods^5,6^ profile the taxonomic composition of metagenomic samples using collections of genomic markers derived from databases of complete and draft genomic sequences. Despite being able to reach strain-level sensitivity^7–9^, these approaches rest on the implicit assumption that a single dominant strain is present for each species, while it has been shown that the human associated microbiota is often a complex mixture of closely related strains of the same species^10^. In these cases, marker-based methods might predict chimeric strains resulting from the overlap of unrelated sequences. As an alternative to using presence–absence profiles, one reference-free method that uses polymorphism patterns in a set of universal marker genes has recently been introduced^11^, providing useful insights in the case of poorly characterized species, where reference genomes are not available. However, to achieve its best performances, this method needs that large data sets of related samples (as, e.g., in time series) are analyzed. Recently, statistical methods able to disentangle mixture of strains from the same species by modeling the measured distribution of sequence reads have been proposed^12,13^. In these methods, no attempt has been made to provide a connection with the population structure of the relevant bacterial species, thus limiting their use in the epidemiology of known, potentially pathogenic species. In addition, the lack of a preprocessing step of the reference genomes might have an impact on their resolution when the reference database contains many closely related sequences.

Here we present StrainEst, a novel, reference-based method that uses the single-nucleotide variants (SNV) profiles of the available genomes of selected species to determine the number and identity of coexisting strains and their relative abundances in mixed metagenomic samples. Rather than providing a general tool that characterizes all species at the same time, StrainEst concentrates on species of interest by defining their population structure through a clustering of the SNV profiles. By using a penalized optimization procedure to disentangle the individual components, StrainEst identifies and quantifies all the strains of the species of interest present in a sample, improving the resolution of current strain identification methods. In addition, by classifying these components using a pre-defined database of representative genomic sequences, StrainEst allows the compilation of large meta-analyses, including samples from unrelated studies and poses the basis for the widespread use of metagenomics in epidemiological studies.

## Results

### Preprocessing of genomes and database preparation

**Fig. 1:**
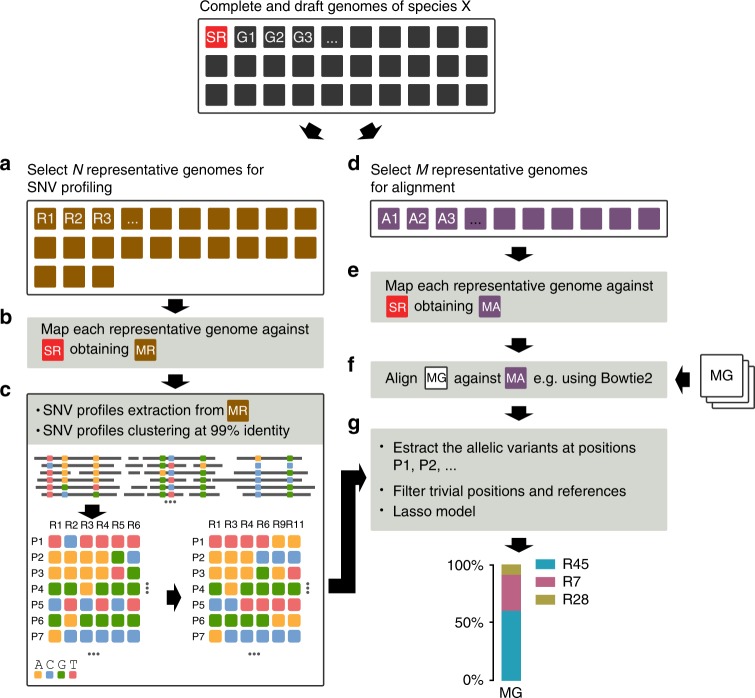
StrainEst overview. **a** Given the complete and the draft genomes of the species of interest (G1, G2,…) and the species representative (SR), the pairwise Mash distances are computed. Genomes with Mash distances >0.1 from the SR are discarded and the remaining ones are clustered to remove redundant sequences. For each cluster, the genome with the lowest average distance from the other members is chosen as a representative (R1, R2,…). **b** The representative sequences are mapped using nucmer against SR and ambiguous mappings are removed. **c** For each representative, the positions of the variant sites (P1, P2,…) are identified and the SNV profiles are extracted. The profiles are clustered at 99% identity to guarantee their representativeness. **d** To create a reference set for metagenomic reads alignments that takes into account the variability of the species, representative genomes are selected for the metagenome alignment step (A1, A2, …) and (**e**) mapped against SR. **f** For each metagenome (MG), the reads are aligned to the chosen genomes using Bowtie 2. **g** The frequencies of the allelic variants at the variant positions defined in step (**c**) are extracted from the BAM file; sites with low coverage are filtered according to user-defined filtering parameters; the relative abundance profile is finally inferred by Lasso regression

For each species of interest, we downloaded all the available complete and draft genome sequences from the NCBI database (see Fig. 1a and Methods section). To eliminate spurious sequences, the genome database was filtered discarding those sequences too divergent from the NCBI type strain of the species and clustered to reduce the redundancy of closely related genomes (see Methods section).

### Computing the core genome and the reference SNV profiles

We aligned each representative genome against the complete genome of a species representative (SR). SR was usually the reference strain on the NCBI website, although other choices are possible (Fig. 1b). During the alignment ambiguous mappings (i.e., those regions that can be mapped in more than one positions in the reference) were discarded. Then, the SNV profiles were extracted from the core genome. Therefore, for each sequence, an ordered vector of SNVs was defined, and the collection of these vectors was the SNV matrix of the species. To define the reference set for subsequent analysis, the SNV profiles were clustered using a complete linkage hierarchical clustering with an identity threshold of 99%. This step ensured that the base strains were sufficiently diverse to allow the optimization step to distinguish them in the mixed samples (Fig. 1c). This procedure yielded a SNV profile for each representative strains (clustered SNV matrix) that was the reference for the following modeling step. The number of representative profiles depended on the genomic variability of the species and on the number of available genomes. For instance, in the case of *Propionobacterium acnes* this was composed by 20 SNV profiles, while in the case of *Escherichia coli* this included 278 reference SNV profiles. See Methods section for more details on the procedure.

### Metagenome alignment and abundance profiles

Given a metagenomic sample, the raw reads were aligned against a set of genome sequences that were chosen in order to be representative of the genomic variability of the species (Fig. 1f and Methods section). For the SNV positions identified previously, we extracted from these alignments the frequency of occurrences of each of the four possible alleles from the aligned reads of the metagenome (Fig. 1g). Then, positions were filtered to remove low coverage sites (see Methods section). Finally, the frequency profile of each metagenome was modeled as a sparse linear combination of the reference SNVs profiles using Lasso regression^14^, where the shrinkage coefficient was optimized using a cross-validation approach. The result of this step was a list of the reference strains and of their relative frequencies that best explained the observed distribution of the allelic frequencies.

### Validation and comparison with existing tools

**Fig. 2:**
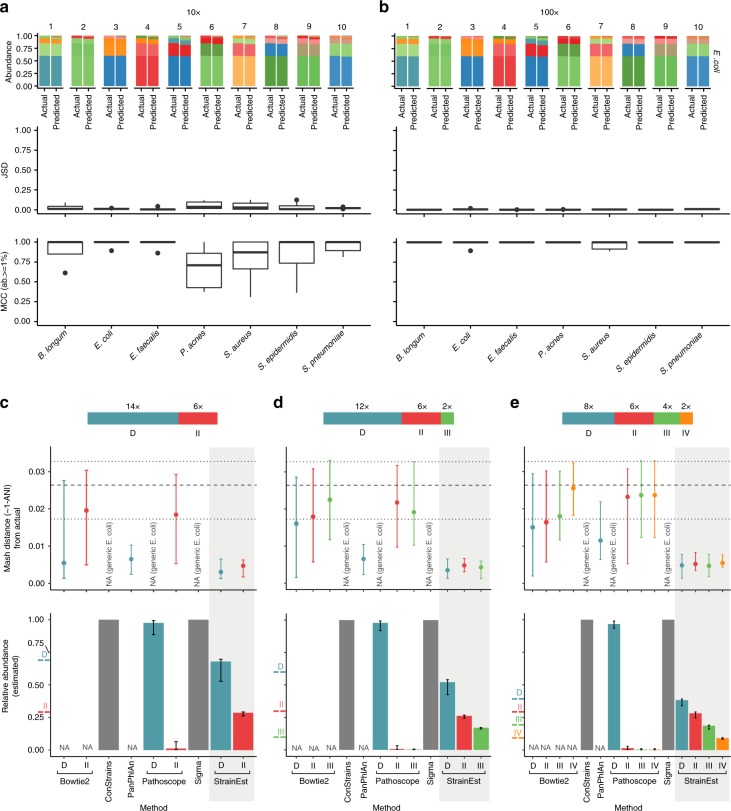
Validation on synthetic data and comparison with existing tools. StrainEst is able to predict the relative abundances of multistrain synthetic mixtures for different species such as *B. longum*, *E. coli*, *E. faecalis*, *P. acnes*, *S. aureus*, *S. epidermidis*, and *S. pneumoniae*. For each species, we simulated 10 synthetic data sets at coverage 10X (**a**) and 100X (**b**) generating reads from four strains mixed at variable relative abundances (60-25-10-5%). In the upper panel, we show the comparison between real and predicted relative abundances for *E. coli*. Colors indicate different strains. In the middle panel, we show the JSD between actual and predicted strain composition. In the lower panel, we show the MCC between the real and predicted strain composition, discarding strains with predicted relative abundances below 1%. As expected, the accuracy of StrainEst grows with increasing coverage. Boxes extend to the first and third quartile, whiskers extend to the upper and lower value within 1.5*IQR from the box. Outliers are shown as points. **c**–**e** Upper panels: distance between the dominant (D) and the second (II), third (III), and fourth (IV) most frequent strain predicted by Bowtie 2, ConStrains, PanPhlAn, PathoScope, Sigma, and StrainEst for the three synthetic data sets composed of 2, 3, and 4 strains of *E. coli*. NA (generic *E. coli*) indicates that the algorithm only predicted the presence of *E. coli* without further specification. The broken lines indicate the 25th percentile, median, and 75th percentile of the distribution of the pairwise Mash distances between pairs of strains randomly chosen from the 3041 *E. coli* genomes downloaded from NCBI. Lower panels: Predicted relative abundances of the identified strains. The expected relative abundances are marked in colors (D, II, III, and IV for the dominant, second, third, and fourth strain in terms of relative abundances, respectively) on the vertical axes. Error bars indicate the first and third quartile

To validate the approach, we generated two sets of synthetic microbiomes, *syntheticII* and *syntheticIV*, using the ART^15^ simulator. In *syntheticII*, we mixed synthetic reads from pairs of genomes of the same species for four bacterial species, namely *Bifidobacterium longum*, *Enterococcus faecalis*, *Staphylococcus aureus* and *Staphylococcus epidermidis* for four different values of the total coverage (10×, 20×, 50× and 100×) and for three different values of the relative abundances (50–50, 70–30 and 90–10%). For each combination of coverage and relative abundance 50 independent samples were generated, for a total of 600 paired-end Illumina HiSeq-2000 metagenomic samples for each species. In *syntheticIV*, 10 independent samples for two different values of the coverage (10× and 100×) were generated by mixing synthetic reads generated from four different genomic sequences of the same species for seven different bacterial species, namely *B. longum*, *E*. *coli*, *E. faecalis*, *P. acnes*, *S. aureus*, *S. epidermidis* and *Streptococcus pneumoniae*. The performances of the algorithm on *syntheticII* were quantified by measuring the absolute deviations between the predicted and true relative abundances of the major and minor components (Supplementary Fig. 1). For all species and values of coverage, we found a good agreement between predictions and true values, with the higher discrepancies at low coverages in cases where the minor component accounted for 10% of the reads, often below the threshold of detection. To further quantify the ability of StrainEst to identify the strains present in the samples, we considered as present the strains with a predicted abundance exceeding a given threshold, and computed the Matthew Correlation Coefficient^16^ (MCC) with the true values as a function of this threshold. For all species (Supplementary Figs. 2–5), the MCC was close to 1 for thresholds exceeding 2% (i.e., when strains with predicted relative abundance below 2% were discarded) and dropped for the mixtures 90–10% when the cutoff threshold exceeded the relative abundance of the minor component. The ability of StrainEst to disentangle complex communities of closely related strains was confirmed by the analysis of the *syntheticIV* data sets (Fig. 2 and Supplementary Fig. 6), where we found that in all cases the strain distribution was reconstructed with high precision, with an average MCC >0.96 (strains with relative predicted abundance below 1% were not considered in the comparison) and an average Jensen–Shannon divergence (JSD) < 0.02 for a total coverage of 100X (Supplementary Tables 1 and 2).

One of the major challenges of reference-based methods is when a strain is missing from the reference database. To show the performances of StrainEst in these cases, we adopted a leave-one-out approach, by selectively removing one reference profile from the *E. coli* database and using this reduced database to characterize one synthetic sample containing only the removed strain (*LOOEcoli* data set). In those cases, StrainEst with default parameters always identified a closely related strain as dominant, but overestimated the sample complexity (Supplementary Fig. 7). Using a more stringent cutoff of the compatibility parameter that defines the profiles that are used in the modeling step (see Methods section) the predictions of the sample complexity progressively improved, while the number of samples that could not be classified increased (Supplementary Fig. 7).

We tested the algorithm on the two mock communities available from the Human Microbiome Project^17^, containing 21 known organisms with even (SRR172902) or staggered composition (SRR172903). For these communities, we tested the ability of StrainEst to identify the correct strain of *E. coli*, *N. meningitidis*, *P. acnes*, *S. aureus*, and *S. epidermidis*. This test was especially challenging, due to the low abundance of some of these species in these samples (see Methods section and Supplementary Table 3). For this reason, we included in the modeling step all SNV positions with coverage ≥1 (the default cutoff for this option is 6). For all species StrainEst identified correctly the presence of one dominant strain that coincided with the strain known to be present, with the exception of *N. meningitidis* in sample SRR172903, where the algorithm did not reach convergence due to the low number of reads that aligned to the reference genomes and the low coverage of SNV sites (Supplementary Table 3).

To highlight the potentialities and the novel features of StrainEst, we compared to PanPhlAn^7^, MIDAS^8^, PathoScope^12^, Sigma^13^, ConStrains^11^ and simple read alignment on reference genomes using Bowtie2^18^ (Fig. 2c–e). PanPhlAn and MIDAS, despite being able to determine the taxonomic structure of metagenomic samples with strain-level resolution were not specifically designed to disentangle mixtures of multiple strains of the same species into single components, and provide no information on the existence and identity of other strains beside the dominant one. Rather, PanPhlAn is able to identify and classify the dominant strain of each species using a reference database, while MIDAS provides the SNV profiles and genomic repertoires of each of these dominant strains (Supplementary Table 4). Thus, MIDAS can be used to assess whether the same dominant strain is conserved across samples (allowing, e.g., the identification of transmission networks), but does not provide data directly comparable to StrainEst due to the lack of a post-processing classification tool. PathoScope and Sigma identify the most likely mixture of source genomes from reads distributions using a penalized mixture model and a stochastic model of read sampling, respectively. ConStrains uses methods from information theory to identify the mixture of strains present in a set of samples from SNV profiles in universal marker genes balancing model fitness and complexity. To quantitatively compare the performances of StrainEst, PanPhlAn, PathoScope, Sigma, ConStrains and Bowtie 2 in the identification of the dominant and subdominant strains, we generated 50 synthetic microbiomes containing 2, 3 and 4 randomly selected strains of *E. coli* using ART with coverage 20× *syntheticEcoli* data set). We found (Fig. 2c–e and Supplementary Data 1) that in all cases ConStrains and Sigma correctly identified the species, but gave no information concerning the presence of different strains. In the majority of cases this was also true for PathoScope, which only in a few cases could identify the presence of multiple strains below the species level. However, the genetic distance between the strains predicted by PathoScope and the actual ones was usually high (see upper panels in Fig. 2c–e). In addition, even when strains below the species level were identified, PathoScope found that the majority of the metagenomic reads supported generic *E. coli*, while the relative abundances of the single strains below the species level were always low. On the other hand, as mentioned before, PanPhlAn could not predict the relative abundances of the different strains, but could identify the dominant one with good accuracy, as measured by the Mash^19^ distance between the predicted and the actual strain. However, the dominant strains predicted by StrainEst were more closely related to the real ones than those identified by PanPhlAn (*P* = 8.06×10^−3^ for the two-strain data set, Wilcoxon rank-sum test), in particular for those cases where the contribution of subdominant strains was higher (*P* = 2.37×10^−5^ for the four-strain data set). In all cases, StrainEst could also identify the subdominant components with high accuracy (Fig. 2c–e, lower panels). In order to show the advantages of using this approach to naive read assignment, we aligned all the reads to the same reference genome database used by StrainEst with Bowtie 2, and selected the most frequent 2, 3, and 4 strains in the three synthetic data sets, respectively. Ambiguous alignments (reads with mapping quality <10) were discarded. The Mash distances between these sequences and the actual ones, also reported in Fig. 2c–e show that the precision of the dominant strain identification by Bowtie 2 is comparable to PanPhlAn in the case of mixtures of two strains, but with a much higher variability between samples. For more complex mixtures and for subdominant strains, read alignment with Bowtie 2 provide strain identification that is in the best cases only slightly better than random. The command lines used for ConStrains, PanPhlAn, PathoScope, Sigma and Bowtie2 are available in Supplementary Methods.

### Diversity and stability of P. acnes in human skin

**Fig. 3:**
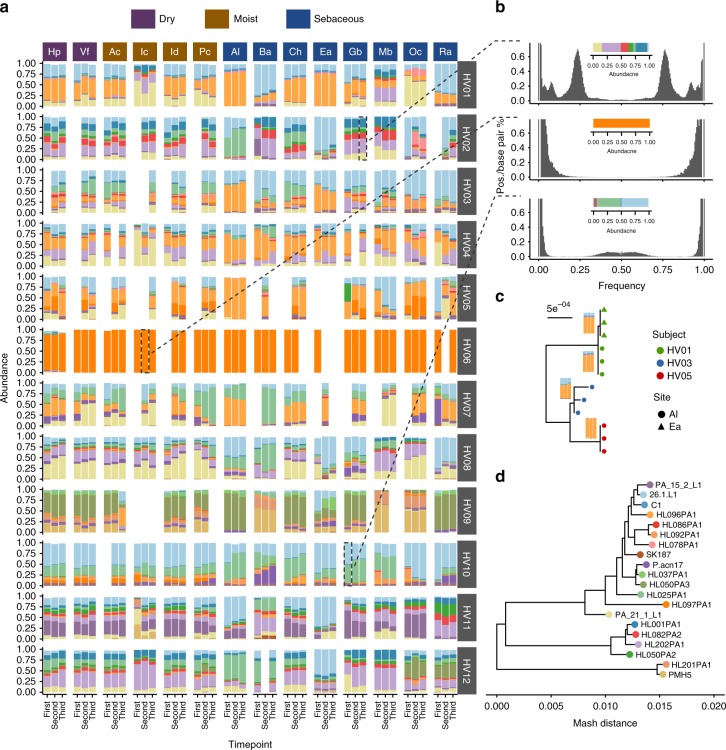
StrainEst analysis reveals interpersonal and intersite differences in the strain composition of *P. acnes* communities in human skin metagenomic samples. Three skin samples from 14 sites from a cohort of 12 healthy subjects were collected at three different times separated by long (1–2 years between timepoints 1 and 2) and short (2–3 months, between timepoints 2 and 3) time intervals. **a** Each individual is colonized by a specific mixture of strains. The relative abundances of the subject-specific mixture vary across the different body sites, but are conserved across the different sampling times. The site codes are described in the original work. The strain identifiers are reported in (**d**). **b** To verify that complex strain mixtures are not an artifact due to the presence of one strain not represented in the collection of genomic reference sequences, we show for three representative samples the distribution of frequencies of the four possible nucleotides at each allelic position. **c** Where a single strain was dominant, we could use the consensus (containing the most supported allele in each position) SNV profile to compare the strains from different subjects/body site. In this example, strains were classified as HL096PA1 by StrainEst cluster by subject and body site. The variability between profiles from subject HV03 is probably due to the lower relative abundance of the dominant components (see also a). In this case, it is likely that the presence of a second strain with nonnegligible relative abundance introduces a source of noise in the consensus SNV profile. **d** Neighbor joining tree of the reference strains. Leaves are colored using the same schema as in (**a**)

In a recent report^10^, it has been shown that *P. acnes* is one of the major components of the skin microbiome, usually forming complex, host-specific communities composed by multiple strains. We have re-analyzed the human skin data set using 110 genomic sequences of *P. acnes* available at NCBI as reference database. After the clustering procedure, 20 distinct references were obtained (Supplementary Fig. 8). We found that each subject was colonized by a different combination of strains, and that while the strains colonizing the different body sites of a given subject were the same, these were mixed in different proportions in different body sites (Fig. 3a) and stable over time. To confirm that these complex patterns were not an artifact due to the presence in the samples of strains not represented in the reference collection of sequenced genomes, for a selection of typical single-, double- and multiple-strains samples, we measured the distribution of site-specific frequency of the four possible allelic variants found for each SNV site (Fig. 3b). In agreement with the strain pattern reconstructed by StrainEst, we found that for samples predicted to harbor a single strain the distribution was bimodal, with allelic variants either supported by 100% of the reads or not present. For double strains samples, two intermediate peaks were found in the distribution, representing those sites where the two strains had discordant alleles. Given that the SNV matrix has been clustered to 99% identity to define the reference profiles (see Methods section), in a sample containing two strains these should account for at least 1% of the sites. Finally, multiple strain samples were characterized, as expected, by complex distributions of the relative frequency of allelic variants. In cases where one single strain was clearly dominant, we used the consensus (i.e., supported by the highest number of reads) SNV profiles to probe its inter- and intra- subject variability (Fig. 3c). Profiles assigned to the same reference strain clearly clustered by subject and intra-subject variability was related to the amount of noise introduced by subdominant strains (e.g., subject HV03 in Fig. 3c).

**Fig. 4:**
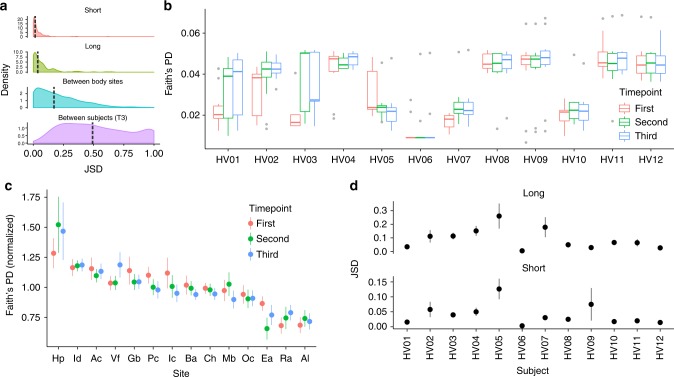
Diversity and richness of *P. acnes* in the human skin data set. **a** Short: for each subject/site pair, we computed the distribution of the JSD between the second and the third time point. Long: the same distribution computed between the first and the second time point. Between body sites: the JSD distribution computed between body sites for each subject/time point pair. Between subjects (T3): for each site, the distribution was computed between subjects at the third time point. Vertical dashed lines represent the median values. **b** Using the predictions of StrainEst, we could give an estimate of the diversity of the subject-specific *P. acnes* populations using Faith’s phylogenetic diversity (PD) index. Two different phenotypes could be identified, with high (HV04, HV08, HV09, HV11, and HV12) and low (HV07 and HV10) PD. Three individuals (HV01, HV02, and HV03) switched between low and high phenotype during the course of the study, while one (HV05) switched from high to low PD. Boxes extend to the first and third quartile, whiskers extend to the upper and lower value within 1.5*IQR from the box. Outliers are shown as points. **c** Faith’s PD computed for each site and normalized per subject highlights more diverse (such as Hp) and less diverse environments (Ea, Ra, and Al). **d** Long: JSD between samples from each individual between the first and second timepoints. Short: the same, between the second and the third timepoints. This individual-specific temporal variability analysis of the *P. acnes* population shows that subjects (e.g., HV01) that are stable in the first interval tend to maintain these characteristics also in the second, while individuals that are characterized by high variability (e.g., HV05) in the first interval are highly variable also in the following time frame. For **c** and **d**, points indicate the mean values, error bars the standard errors

Using the relative abundances predicted by StrainEst, we could show that the populations of colonizing strains of *P. acnes* were more similar between samples from the same subject, same body site at different times, than between samples from the same subject and different body sites, and different subjects (Fig. 4a). The per-subject Faith’s phylogenetic diversity^20^ (PD) of the *P. acnes* populations identified two classes of individuals, one with low and one with high diversity (Fig. 4b). In three individuals, we could monitor a switch from the low to the high diversity phenotype (HV01, HV02 and HV03) during the course of the study (see also Supplementary Fig. 9), and one from the high to the low diversity phenotype (HV05). Three of these four individuals were defined as “high variable” for the general instability of their microbiota in the original study^10^. Looking at the different sites across individuals, we found that there was a wide distribution of diversity of the colonizing population of *P. acnes* with the most diverse site being the hand palm that had a PD double than what found on alar and retroauricular crease (Fig. 4c). Finally, we found that individuals that had a highly dynamic *P. acnes* population between the first two time points (Fig. 4d, upper panel) also had larger distances between the second and the third time points (Fig. 4d, lower panel), suggesting that the overall stability of the *P. acnes* population is a subject-specific characteristics.

### Spatial distribution and diversity of oral Neisseriae

**Fig. 5:**
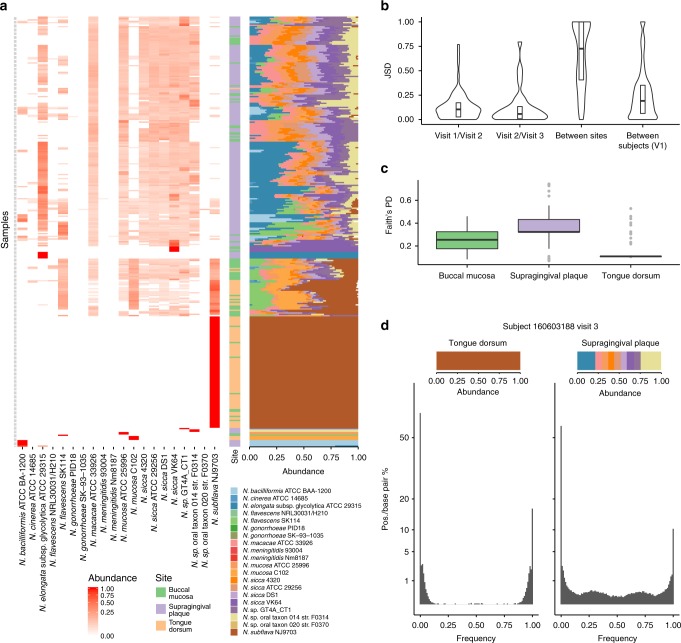
StrainEst disentangles complex mixtures of neisseriae in 320 oral samples from the HMP. While tongue dorsum samples are dominated by *N. subflava*, the other two sampling sites, namely the supragingival plaque and the buccal mucosa are characterized by much more complex communities (**a**) and (**c**). Samples are ordered by an average linkage hierarchical clustering (Bray–Curtis dissimilarity). The Jensen–Shannon divergence (**b**) is significantly higher between sites or between subjects than between visits in the same site/subject (*P* < 0.001), suggesting that the population of *Neisseria* strains is stable over an extended period of times. **d** Distribution of site-specific frequencies of the four possible nucleotides at each allelic position for two representative samples from the same subject. As in Fig. 1b, single-strain samples have a distribution with two peaks at frequencies close to 0 and 1. Complex communities are characterized by symmetric distributions with multiple peaks at intermediate values. In **c**, boxes extend to the first and third quartile, whiskers extend to the upper and lower value within 1.5*IQR from the box. Outliers are shown as points

Core genome-based approaches, like StrainEst, are particularly suited for the analysis of metagenomic samples below the level of species. However, in the case of closely related species with similar genomes, a general framework to analyze the data across the different species can be set up. To show the use of StrainEst both between and within species, we consider the many species from the *Neisseria* genus that can colonize the human oral cavity. In a recent study^21^, it was shown that different species dominate the samples from different sites in the oral cavity with a variable degree of strain admixture, leaving the open question concerning the real, site-specific population structure of this group of species, i.e., how many individual strains/species were present in each sample. To verify if, besides the dominant component, samples from the different sites contained complex mixtures of other species and the site-specific structure of these communities, we analyzed 689 oral samples from the Human Microbiome Project, using a database of 212 genomic sequences of human associated neisseriae. The core genome of these species contained 25.393 SNV positions and the genomes were grouped into 79 reference clusters by the StrainEst pipeline (Supplementary Table 5). We found striking differences amongst three main oral sampling sites of the HMP, i.e., the tongue dorsum, the supragingival plaque and the buccal mucosa (Fig. 5 and Supplementary Fig. 10). While the majority of samples from the tongue dorsum contained exclusively *Neisseria subflava*, most samples from the other two sites appeared to contain much more complex mixtures of species. Interestingly, there was a clear distinction between the tongue dorsum and the supragingival plaque, and the buccal mucosa was intermediate between the two, with a fraction of the samples clustering together with samples from the tongue dorsum and a smaller fraction clustering with samples from the supragingival plaque. The former were characterized by the presence of *N. subflava* that was almost completely absent from the supragingival plaque. These findings were reflected in the phylogenetic diversity of the *Neisseria* population of samples from the three sites, that was significantly higher in the supragingival plaque than in the buccal mucosa (FDR corrected *P* = 1.2×10^−6^, Wilcoxon rank-sum test), and in the tongue dorsum (*P* = 3×10^−7^). An analysis of the Jensen–Shannon Divergence showed that the diversities between the reconstructed populations of neisseriae were significantly lower between the same sites and subjects sampled at different times (average time between visits of 219 days, s.d. of 69 days) than between sites or between subjects (*P* < 0.001 for both comparisons), suggesting that the same mixture of strains is retained in each individual site for extended periods of time.

### Escherichia coli strains in cross-sectional studies

**Fig. 6:**
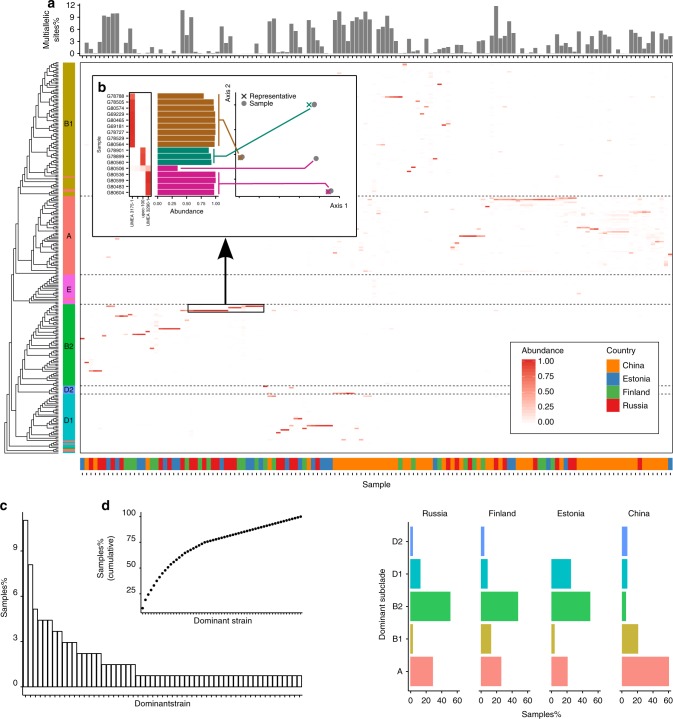
*E. coli* meta-analysis. **a** Distribution of *E. coli* strains in two large studies including fecal samples from 222 infants from Estonia, Finland, and Russia, and 345 adults from China. Samples with a reconstruction Pearson R < 0.9 and a minimum depth of coverage < 10 were discarded obtaining a total of 136 individuals. The upper panel reports the percentage of sites where the dominant allelic variant is supported by less than 90% of the aligning reads, suggesting the presence of more than one strain. The origin of the sample is shown by the lower bar. Samples are ordered by an average linkage hierarchical clustering using weighted UniFrac distance. **b** Consensus SNV profile from samples dominated by four closely related strains is clearly distinct and closely related to the reference strain identified by StrainEst. In one case (sample G80506), StrainEst fails to identify the dominant strain, probably due to the lack of a closely related reference in the sequence database. Considering only the dominant component, only 23 strains were sufficient to cover 75% of the samples (**c**, **d**). Despite the presence of several ubiquitous strains, clustering of the samples according to their origin was evident. This clustering was related to the prevalence of the different phylogroups, shown in **e**. While the dominant strain was in 60.3% of the cases from phylogroup A in the Chinese panel, this percentage was 20.8%, 26.1%, and 29.3% in Estonian, Finnish, and Russian infants, respectively. In the latter samples, the most frequent dominant strain was in all cases from phylogroup B2 (50.0%, 47.8%, and 51.6%)

Amongst the advantages of the reference-based approach of StrainEst there is the possibility to perform large-scale meta-analyses and comparisons of unrelated studies, and to use the data for molecular epidemiological surveys for species of particular relevance, analogously to what is done using the popular Multi Locus Sequence Typing (MLST) schema. To illustrate the potentialities of StrainEst in epidemiological studies, we have applied the method to determine the strain distribution of *E. coli* in two recent large metagenomic studies from stool samples. *E. coli* is an extremely diverse species with a large variability between strains that can range from beneficial intestinal commensals to aggressive extraintestinal pathogens. Population genetics studies based on genetic markers^22^ and whole-genome comparative analysis^23,24^ have identified six phylogenetic groups, namely A, B1, B2, D1, D2 and E, associated to different ecological niches and showing variable pathogenic potential. The StrainEst reference database was built using 3041 complete and draft genomic sequences downloaded from the NCBI database. After the clustering procedure, these were grouped into 278 distinct reference clusters. The representative sequences of these clusters were assigned to one of the six phylogenetic groups according to their Mash distance from the reference strain of each group, namely *E. coli* str. K-12 substr. MG1655 for A, *E. coli* O104:H4 for B1, *E. coli* O83:H1 str. NRG 857C for B2, *E. coli* UMN026 for D1, *E. coli* IAI39 for D2, and *E. coli* O157:H7 str. Sakai for E. The profile of SNVs included 104248 allelic sites. Using this reference database, we analyzed two large metagenomic data sets from stool samples, one from 222 infants in Estonia, Finland and Russia^25^, and the second from 345 adult Chinese individuals^26^. The percentage of multiallelic SNV sites ranged from 0 to 11.8%, showing evidence that several samples were colonized by more than one strain (Fig. 6a). Concentrating on a subset of samples dominated by closely related strains, we found that in all cases the consensus allelic profile of the metagenomic samples was closely associated to the reference strain (Fig. 6b). In one case (sample G80506), StrainEst predicted a complex pattern of strains, that was not supported by the percentage of multiallelic sites (Fig. 6a). In this case, a single strain clearly distinct from all the reference strains was present (Fig. 6b). Strikingly, the population of circulating strains of this diverse species could be described by a relatively small number of ubiquitous strains that were often present in individuals from all the four distinct populations. The set of strains that dominated at least one sample in our meta-analysis included only 57 strains out of the 278 that represented the full pool of sequenced *E. coli* isolates (Fig. 6c). However, the different populations were clearly distinguishable by the mixture of strains colonizing them. If we consider only the dominant strain for each sample, the most common strains in the samples from the Chinese adults were from phylogroup A, while the most common phylogroup in the infants from Russia, Estonia and Finland was B2 (Fig. 6e).

### Time series during infant gut colonization

**Fig. 7:**
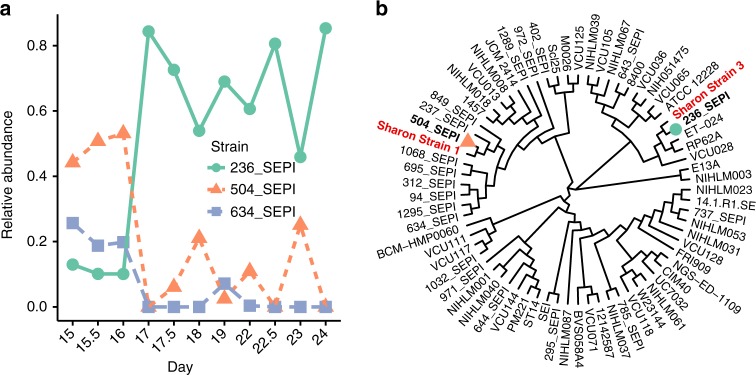
S. *epidermidis* strains in early stages of an infant gut colonization. **a** Three coexisting strains were found, with shifting relative abundances. While the three initial samples were dominated by strain 504_SEPI, at later times, strain 236_SEPI was the most abundant. **b** Whole-genome cladogram of the reference genomes, also including the two high-quality genome sequences (Sharon strain 1 and Sharon strain 3) assembled in the original paper from the same samples. These genomes are closely related to the two strains identified by StrainEst

A time series study of infant gut colonization has shown that multiple strains of a limited number of species dominate the infant gut during early stages of life^27^. In particular, it was shown that in a premature infant three strains of *S. epidermidis* were present and changed considerably their relative abundances over time. In the original study, two high-quality genomes and one partial genome of *S. epidermidis* were assembled from these samples. We have analyzed the data from^27^ to identify the strains of *S. epidermidis* present in the samples and determine their relative abundances. We found that we can confirm the presence of three strains, with large shifts of their relative abundances (Fig. 7a). In particular, while at the beginning of the sampling period we found that a strain closely related to strain 504_SEPI was dominant, at later times there was a switch to strain 236_SEPI, confirming the findings of the original publication^27^. Comparing the two high-quality genomes assembled in^27^ to the genomes of the strains identified by StrainEst, we found that these are the most similar in the reference database of complete and draft genomes (Fig. 7b).

## Discussion

Metagenomic technologies are rapidly becoming the major source of information concerning microbial ecology. To fully exploit their potential, data analysis techniques able to disentangle potentially complex communities of closely related strains and to classify them according to the population structure of the species are needed. In many cases, species of clinical or biotechnological relevance have been extensively studied and large databases of genomic sequences from single isolates exists, together with a large body of knowledge on the individual characteristics of selected strains. StrainEst is a method to determine the strain composition of complex metagenomic samples for species for which sufficient genomic information are available. While being an obvious limitation in the case of poorly characterized species, the reference-based approach encoded in StrainEst has the advantage of providing a classification of the strains in term of a standard set of representative sequences whose number and identity is derived from the population structure of the species. SNV profiles have already been used to analyze metagenomic samples and to demonstrate the temporal stability of the individual microbiota in human hosts^10,28^, but general methods to use their distribution to identify individual strains and quantify their relative abundances have not been proposed yet. Two major points differentiate what we have done from previous approaches: (i) we explicit the definition of strains as clusters of similar sequences, and we provide a computational pipeline to define the reference sequence database, also allowing the user to choose its own definition of the cluster radius; (ii) we use a penalized optimization procedure to guarantee that the SNV patterns are explained using the minimal amount of independent strains. The latter step is in particular key to use the SNV data to answer the important biological question if more that one strain of a given species is present in a sample, and, if yes, how many they are and what are their relative abundances. Without the penalization step, overfitting might lead us to overestimate the complexity of samples where a single strain is present, that by chance belongs to a group where many very similar reference genomes are available, leading us to erroneously conclude that a complex population is present. In this situation, a comparative analysis of the diversity of unrelated samples would be impossible because the results would be biased by the number of genomes present in the database for each particular group. Instead, the preliminary definition of the reference database and the use of a penalized linear model guarantee that only the minimum set of clearly distinct reference genomes is used in the modeling step. This allows on one hand the portability of the data across different studies, and on the other the interpretation of the data using the accumulated body of knowledge concerning the epidemiology, distribution, pathogenic potential and individual characteristics of the reference strains, posing the basis for the widespread use of metagenomics in epidemiological studies.

The classification provided by StrainEst is based on strain-specific characteristics of the core genome of a species, and as such is not intended to identify features in the dispensable genome that might nevertheless be important in determining the individual characteristics of a strain, like, for instance, pathogenicity islands or antibiotic resistance genes^23,29^. It has long been known that some species are more recombinogenic than others^30,31^, and that this corresponds to different degrees of correlation between core and dispensable genome. StrainEst, like all molecular typing schema based on conserved marker loci, relies on the degree on genetic linkage between these and the features of interest. If this is not sufficient, more specialized, feature-specific methods need to be applied.

## Methods

### Databases preparation and metagenome read alignment

The StrainEst pipeline uses a set of genome sequences to compute reference SNV profiles as the basis for the modeling step and a second set (usually smaller than the previous one to reduce the computational burden) of genomes as a target for metagenomic read alignment. The selection of these two reference genome databases should be done by the user according to its needs and goals. The procedure sketched below is adapted for exploratory studies where the epidemiology of a given species is studied. For more focused studies, like, for instance, when the dynamics of a restricted set of strains is studied in time series, other strategies are possible.

### Representative genomes for the SNV profiling

In this step, we selected the representative set of genome sequences to calculate the SNV profiles that are the basis of the modeling step and define the resolution of the method. For each species of interest, we downloaded all the available complete and draft genomes from the NCBI web site. For each species, the reference genome on the NCBI web site was chosen as the species representative (SR, see Fig. 1), namely *P. acnes* str. KPA171202, *N. meningitidis* str. MC58, *E. coli* str. K-12 substr. MG1655 for *P. acnes*, *N. meningitidis*, and *E. coli*, respectively, in the examples presented here. Given the complete and draft sequences (G1.fasta, G2.fasta,…) of the species of interest, the pairwise Mash distances (≈1−*ANI*) were computed using the Mash software, a recent alignment-free tool that can provide a complete pairwise distance matrix for large-sequence data sets, avoiding the need of computing pairwise whole-genome alignments, a step that is computationally demanding in large data sets. The command line for computing the Mash distance was

mash sketch -o sketch G1.fasta G2.fasta

mash dist -t sketch.msh sketch.msh>mash.dist

Genomes with Mash distances >0.1 ( >0.2 for the neisseriae, where sequences from closely related species were included in the reference database) from SR were discarded. This choice of the distance threshold guaranteed that unrelated genomes were excluded, while divergent strains from highly variable species were retained. For comparison, the bacterial species definition threshold was set to 0.05 in the original Mash paper^19^ (95% ANI).

A complete linkage hierarchical clustering using the distances computed in the previous step was then performed using a threshold between 0.001 and 0.006 for the different species (approximately an ANI from 99.9 to 99.4%, respectively, Supplementary Table 6). Given that there is an inverse relationship between the number of sequences that are included in its definition and the size of the core genome due to the fragmented nature of the majority of the genomes available on public databases, the threshold was chosen as the lowest value below which the core genome of the species fell below 10% of the length of the species representative genome. For each cluster, the genome with the lowest average distance from the members of its group was chosen as a representative (R1, R2,…, R*N*, see Fig. 1a). Other clustering strategies could be used in this step, that adjust a local cluster radius according to the sequence variability of each group of sequences by optimizing a measure of clustering quality^32^. However, complete linkage hierarchical clustering was a convenient choice for this step, since it allowed us to control explicitly the maximum distance between each sequence and its representative, thus giving full control over the precision by which a genome sequence is approximated. After clustering, highly similar, closely related isolates and resequencing of strains were represented by a single genome.

### Representative genomes for the metagenome alignment

Given the short length of sequencing reads, aligning metagenomes against a single reference could introduce a bias toward strains closely related to the reference. To make the method more robust, we generated a more comprehensive reference set including 10 aligned genomes representative of the genomic variability of the target species. While the number of reference genomes to include in this step depends on the genomic variability of the species and should be tuned accordingly, we found that using 10 reference genomes that we could align over 80% of the sequencing reads for all species included in the *syntheticIV* data set (Supplementary Table 7), making this choice a good compromise between sensitivity and computational burden. The procedure was the following: i) given the Mash distances computed before, a complete linkage hierarchical clustering was performed and ii) the minimum distance threshold that guaranteed that no more than *M* clusters were formed (10 in the considered cases) was computed; iii) for each cluster, the genome with the lowest average distance from the members of its group was chosen as a representative (A1,…, A10, see Fig. 1d). This procedure guaranteed that the selected sequences provide an even sampling of the species variability also in the case of species with skewed composition of the genome sequence database. This might occur, for instance, in the case of large-scale resequencing of pathogenic strains. However, when some *a priori* knowledge of the microbiota composition is available, more focused choices are possible. For instance, if the dynamics of the long-term colonization of a single individual is studied, the sequences of the isolates that are known to be present in the microbiota, if available, could be used as a reference.

### The StrainEst pipeline

The representative genomes for the SNV profiling (Fig. 1a) defined in the previous step (R1, R2,…) were aligned against the SR using MUMmer^33^ (nucmer command with default parameters), and ambiguous mappings (i.e., regions that can be mapped against more than one region) in alignments were discarded (Fig. 1b). The StrainEst command line was

strainest mapgenomes R1.fasta R1.fasta… SR.fasta MR.fasta

where the file MR.fasta contained the output alignment. The positions that were variable in at least one genome and their allelic variants were recorded in the SNV matrix snp.dgrp (Fig. 1c):

strainest map2snp SR.fasta MR.fasta snp.dgrp

In this matrix, each row corresponded to a variable position in the reference genome and each column contained the allelic variants (A, C, G, and T) of one genomic sequence in those positions. In some cases, pairs of sequences were characterized by vectors of SNVs differing only in few positions, preventing an unambiguous separation of these different components in mixed samples. In order to guarantee that these arrays contained enough information to allow us to distinguish between different strains, we computed the number of sites where the two vectors were different between each pair of sequences, followed by a complete linkage hierarchical clustering with an identity threshold of 99%. The distance matrix was computed using the strainest snpdist command (Fig. 1c):

strainest snpdist snp.dgrp snp_dist.txt

After that, the clustered SNV matrix was computed using the strainest snpclust command:

strainest snpclust snp.dgrp snp_dist.txt snp_clust.dgrp clusters.txt

For each cluster, the SNV profile with the lowest average distance from the other cluster members was chosen as a representative. Fixing an identity threshold below which two SNV profiles fall in the same cluster allows the user to control the resolution for strain identification. This threshold should be determined as the lowest possible that guarantees that a sufficient number of discordant SNV positions exists between two profiles to allow the linear modeling step to distinguish them. In the examples that we have considered, an identity threshold of 99% is equivalent to a number of discordant SNV sites between ~250 for the Neisseriae and ~1150 for *P. acnes* (see Supplementary Table 6) that are sufficient for disentangling complex mixtures. In the case of *E. coli*, the original 3041 sequences downloaded from the NCBI web site were clustered into 278 distinct SNV profiles (see Supplementary Table 6). The SNV profiles were the reference for the final modeling step, and the associated sequences were the representative strains of the species.

The representative genomes for the metagenome alignment (Fig. 1d) defined in the previous step (A1, A2,…) were aligned against the species representative (SR), using the strainest mapgenomes command (Fig. 1e):

strainest mapgenomes A1.fasta A2.fasta… SR.fasta MA.fasta

Then, the Bowtie^18^ index was built:

bowtie2-build MA.fasta MA

The FASTA files used to create the Bowtie 2 database are available at ftp://ftp.fmach.it/metagenomics/strainest/bowtie_fasta/. Given a metagenomic sample (MG), the raw reads were quality trimmed using sickle (https://github.com/najoshi/sickle):

sickle pe -f MG1.fastq -r MG2.fastq -t sanger -o MG1.trim.fastq -p MG2.trim.fastq -s MG.singles.fastq -q 20

The trimmed reads were then aligned against the Bowtie database defined before, and a sorted and indexed BAM file was created:

bowtie2 --very-fast --no-unal -x MA -1 MG1.trim.fastq -2 MG2.trim.fastq -S MG.sam

samtools view -b MG.sam > MG.bam

samtools sort MG.bam -o MG.sorted.bam

samtools index MG.sorted.bam

The Bowtie 2 parameters used above (in particular the --very-fast option) guaranteed sufficient accuracy in all the examples that we have studied. However, in cases in which increased alignment sensitivity is needed, other options are possible, as well as the use of other alignment programs (like, e.g., bwa)^34^. The user should gauge the choice of the alignment program and parameters to the problem at hand. Finally, the strainest est command predicted the strain abundances:

strainest est snp_clust.dgrp MG.sorted.bam outputdir

The strainest est command operates as follows. For the SNV positions $(p_1, \ldots ,p_L)$ identified previously, we extract the frequency of occurrences for each nucleotide (A,C, G, and T) from the aligned metagenome, obtaining the 4*L*-vector of frequencies ***f***:

${\boldsymbol{f}} = \left( {f_{\mathrm{A}}^{p_1},f_{\mathrm{C}}^{p_1},f_{\mathrm{G}}^{p_1},f_{\mathrm{T}}^{p_1}, \ldots ,f_{\mathrm{G}}^{p_L},f_{\mathrm{T}}^{p_L}} \right)^T,$ where $f_{\mathrm{B}}^{p_i} \in \left[ {0,1} \right]$ and *p*_*i*_ is the *i*th allelic position. Similarly, for each reference genome, the SNV profile can be written as

$${\it{r}}_{\it{j}} = \left( {r_{\mathrm{A}}^{p_1},r_{\mathrm{C}}^{p_1},r_{\mathrm{G}}^{p_1},r_{\mathrm{T}}^{p_1}, \ldots ,r_{\mathrm{G}}^{p_L},r_{\mathrm{T}}^{p_L}} \right)^{\mathrm{T}},\; \; \;j = 1, \ldots ,G,$$

where

$$r_{\mathrm{B}}^{p_i} = \left\{ {\begin{array}{*{20}{c}} {1,{\mathrm if}\;{\mathrm the}\;{\mathrm variant}\;{\mathrm is}\;{\mathrm the}\;{\mathrm nucleotide}\;{\mathrm{B}}\;{\mathrm at}\;{\mathrm the}\;{\mathrm position}\;p_i,} \\ {0, {\mathrm otherwise},} \end{array}} \right.$$

and *G* is the number of reference genomes. Therefore, the SNV matrix can be written as a 4*L*×*G* matrix ${\boldsymbol {R}} = \left( {{\it{r}}_1,{\it{r}}_{\mathrm{2}}, \ldots ,{\it{r}}_G} \right)$.

Positions in ***f*** and ***R*** with depth of coverage lower than the 10th percentile and higher than the 90th percentile and in any case lower than 6 (these parameters can be changed by the user) are excluded from the analysis. $f_{\mathrm{B}}^{p_i}$smaller than 0.01 (1% abundance) are set to zero. To reduce the computational burden of the prediction step, only reference genomes that are 95% compatible (*i.e*., 95% of the alleles in its profile are present in the metagenome) are used in the Lasso regression (default parameter). In the case that no reference genome satisfies this condition, no prediction is returned. This guarantees that if the sample contains a strain that is divergent from all the sequences in the reference database, StrainEst will not try to model it as a superposition of reference strains. Rows in the ***R*** matrix that contain only 0 s are discarded. At the end of this filtering step, we obtain the filtered vector of allele frequencies ${\hat{\boldsymbol f}}$ and the filtered SNV matrix ${\hat{\boldsymbol R}}$.

Relative abundances are predicted minimizing the $L_1$ penalized residual sum of squares (Lasso regression^14^):

$${\tilde{\boldsymbol \beta }} = \mathop {{{\mathrm{argmin}}}}\limits_{{\boldsymbol{\beta }} \in {\Bbb R}^G|\beta _k \ge 0} \parallel {\hat{\boldsymbol f}} - {\hat{\boldsymbol R}}\beta \parallel _2^2 + \alpha |{{\beta }}|_1,$$

where the regression coefficients are constrained to be nonnegative. *α* controls the strength of the *L*_1_ penalty and is optimized by a 20× random-subsampling cross-validation (test size 50%) choosing the most parsimonious model within one standard error of the best model (i.e., the model with the lowest mean-squared error). ${\tilde{\boldsymbol \beta }}$ is finally scaled to have a unit norm, obtaining the strain relative frequencies ${\hat{\boldsymbol \beta }} = \left( {\hat \beta _1,\hat \beta _2, \ldots ,\hat \beta _{\it{G}}} \right)^{\it{T}}$. To assess the quality of the prediction, we define the Pearson correlation coefficient between the measured and predicted allelic frequencies as

$$R = \frac{{{\mathop{\rm{cov}}} \left( {{\tilde{\boldsymbol f}},{\hat{\boldsymbol f}}} \right)}}{{{\it{\sigma }}_{{\tilde{\boldsymbol f}}},\sigma _{{\hat{\boldsymbol f}}}}},$$

where ${\tilde{\boldsymbol f}} = {\hat{\boldsymbol R}}{\hat{\boldsymbol \beta}}$ are the predicted frequencies and $\sigma _{{\tilde{\boldsymbol f}}},\sigma _{{\hat{\boldsymbol f}}}$ are the standard deviations of ${\tilde{\boldsymbol f}}$ and ${\hat{\boldsymbol f}}$ respectively.

### Prediction accuracy assessment

In order to measure the accuracy of the predicted strain profiles in synthetic data sets, we computed the Jensen–Shannon divergence and the Matthews correlation coefficient^16^ between the actual and the inferred frequencies.

Given two probability distributions (or relative abundance profiles) *A* and *P*, the Jensen–Shannon divergence is defined as

$${\mathrm{JSD}}\left( {A\parallel P} \right) = \frac{1}{2}D\left( {A\parallel K} \right) + \frac{1}{2}D\left( {P\parallel K} \right),$$

where

$$K = \frac{1}{2}\left( {A + P} \right)$$

and $D\left( {A\parallel P} \right)$ is the Kullback–Leibler divergence from *A* to *P* and it is defined as

$$D\left( {A\parallel P} \right) = \mathop {\sum }\limits_i A(i)\log _2\frac{{A(i)}}{{P(i)}}.$$

The Jensen–Shannon divergence is symmetric and, for two probability distributions, it is in the interval $\left[ {0,1} \right]$^35^.

The Matthews correlation coefficient is a widely used measure to assess the quality of binary classifications. The MCC is defined as

$${\mathrm{MCC}} = \frac{{{\mathrm{TP \times TN}} - {\mathrm{FP \times FN}}}}{\sqrt{{{{\mathrm{(TP + FP)(TP + FN)(TN + FP)(TN + FN)}}}}}},$$

where TP is the number of true positives, TN the number of true negatives, FP the number of false positives, and FN the number of false negatives. The MCC returns a value comprised in the interval$\left[ { - 1,1} \right]$, where 1 represents an exact prediction, 0 a random prediction, and –1 indicates total disagreement between the predicted and actual values.

### *syntheticII* data set

For each species analyzed, we generated 600 Illumina HiSeq-2000 paired-end samples using the ART^15^ simulator. In particular, we took the representative genomes (Supplementary Table 6) of *Bifidobacterium longum* (30 genomes), *Enterococcus faecalis* (264 genomes), *Staphylococcus aureus* (761 genomes), and *Staphylococcus epidermidis* (146 genomes), and for each combination of coverage (10X, 20X, 50X, and 100X) and relative abundance (50–50%, 70-30%, and 90–10%), 50 independent samples were simulated.

### *syntheticIV* data set

For seven different bacterial species, (*B. longum*, *Escherichia coli*, *E. faecalis*, *Propionibacterium acnes*, *S. aureus*, *S. epidermidis*, and *Streptococcus pneumoniae*), we generated 10 independent Illumina HiSeq-2000 paired-end samples for two different values of the coverage (10X and 100X) using the ART simulator. For each sample, reads were generated from four different genomic sequences of the same species randomly selected from the reference genomes in the SNV matrix (see Supplementary Table 6).

### *syntheticEcoli* data set

For the *Escherichia coli* species, we generated 50 independent Illumina HiSeq-2000 paired-end samples (20X of coverage) using the ART simulator randomly picking 2, 3, and 4 genomes from 3041 RefSeq assemblies, for a total of 150 metagenomic samples.

### *LOOEcoli* data set

For the leave-one-out *E. coli* data set, we generated 50 independent samples (20X of coverage) containing one single strain taken from the list of reference genomes in the SNV matrix. Consequently, for each sample, the corresponding reference profile was removed from the SNV matrix and the latter was used by StrainEst to estimate the abundance profile. This procedure (i.e., using only sequences of representative genomes present in the SNV matrix to generate the synthetic data) guarantees that the test is robust to the case of databases with uneven composition, including a large number of closely related sequences and a smaller number representing the genomic diversity of the species. In that case, a leave-one-out experiment on randomly chosen sequences would bias the test toward the more common types.

### ART parameters

The template ART command used for the generation of the *syntheticII*, *syntheticIV*, *syntheticEcoli*, and *LOOEcoli* data sets was

art_illumina -i GENOME, -l 100 -m 350 -s 50 -ss HS20 --fcov COVERAGE --noALN -o OUTPREFIX

Synthetic read samples were mixed to simulate the complete metagenomes.

### HMP mock communities

We downloaded from the NCBI web site two metagenomic samples obtained from two artificial microbial communities containing 21 known organisms with even (SRR172902) or staggered composition (SRR172903). The data sets were analyzed using StrainEst for *E. coli*, *N. meningitidis*, *S. aureus*, and *S. epidermidis*. Due to the low coverage of some of these species (see Supplementary Table 3), the strainest est command was run with the option -a 1, to include in the estimation step all positions with coverage >1. The composition of these samples is available from the HMP web site https://www.hmpdacc.org/HMMC/.

### Oh et al. human skin

A total of 616 samples^10^ were downloaded from the NCBI SRA archive. The data set is composed of 12 healthy subjects and a longitudinal sampling occurred at 10–30 months (“long”) and 5–10 weeks (“short”), and 14 body sites (Hp: hypothenar palm, Vf: volar forearm, Ac: antecubital fossa, Ic: inguinal crease, Id: interdigital web, Pc: popliteal fossa, Al: alar crease, Ba: back, Ch: cheek, Ea: external auditory canal, Gb: glabella, Mb: manubrium, Oc: occiput, Ra: retroauricular crease). “Foot” samples and samples with a prediction *R* < 0.8 were removed and a total of 458 samples were analyzed. The data set is available at the NCBI SRA archive under the study accession SRP002480.

### HMP oral

A total of 689 oral samples^17^ (121 subjects) were downloaded from the HMP web site (http://www.hmpdacc.org/) and analyzed. The oral sites considered in the analysis were the tongue dorsum, the buccal mucosa, and the supragingival plaque. Only samples with a prediction *R* *≥* 0.8 (320 samples in total) were considered in this study.

### *E. coli* meta-analysis

The “three-country cohort” stool samples^25^ were downloaded from the DIABIMMUNE web page https://pubs.broadinstitute.org/diabimmune/three-country-cohort. The data set consists of filtered (using kneadData v0.4) WGS data from 222 infants (33–1163-days old) from Estonia, Finland, and Russia. A total of 345 Chinese adult samples^26^ were downloaded from the NCBI SRA database under the accessions SRP008047 (stage I) and SRP011011 (stage II). Samples with a prediction R < 0.9 and a minimum depth of coverage < 10 were discarded, obtaining a total of 136 individuals (78 infant samples, 58 Chinese adult samples).

### Sharon et al. infant gut

A total of 11 fecal samples collected on postnatal days (15–24)^27^ were downloaded from the NCBI SRA archive under the study accession SRP012558. This data set reports the microbial colonization of the gut of a premature infant born at 28 weeks of gestation.

### Execution time and memory requirements

To assess the performance of StrainEst in terms of execution time and memory requirements, we ran the strainest est command (with one thread) on four samples of the *syntheticII* data set for four different coverages (10X, 20X, 50X, and 100X, see Supplementary Table 8). The running time ranges from 12 min (*S. aureus*, 10X) to 25 min (*S. epidermidis*, 100X) and the maximum memory occupied is in the interval between 129 and 591 MB. The number of iterations required by the coordinate descent algorithm in the Lasso regression and therefore the running time may vary. The tests were run on a desktop machine with an Intel ® Core™ i7-3770, four cores, and 16 GB of RAM.

### Code availability

StrainEst is an open-source, Python-based software. The source code, the documentation, and the reference databases are available at https://github.com/compmetagen/strainest. A self-contained Docker (https://www.docker.com/) image with preinstalled StrainEst, Sickle, and Bowtie2 is available at https://hub.docker.com/r/compmetagen/strainest/.

### Data availability

The *syntheticEcoli* and the *LOOEcoli* data sets are available at ftp://ftp.fmach.it/metagenomics/strainest/synthetic/.

The authors declare that the data supporting the findings of this study are available from the authors upon reasonable request.

## Electronic supplementary material

Supplementary Information

Description of Additional Supplementary File

Supplementary Data 1
