# Supplementary material for: Strain profiling and epidemiology of bacterial species from metagenomic sequencing
Source: Nat Commun. 2017 Dec 22;8:2260. doi: 10.1038/s41467-017-02209-5 (PMC5741664; doi:10.1038/s41467-017-02209-5)
Supplement: Supplementary file 1 — Supplementary Information [file 41467_2017_2209_MOESM1_ESM.pdf]

Supplementary Figures

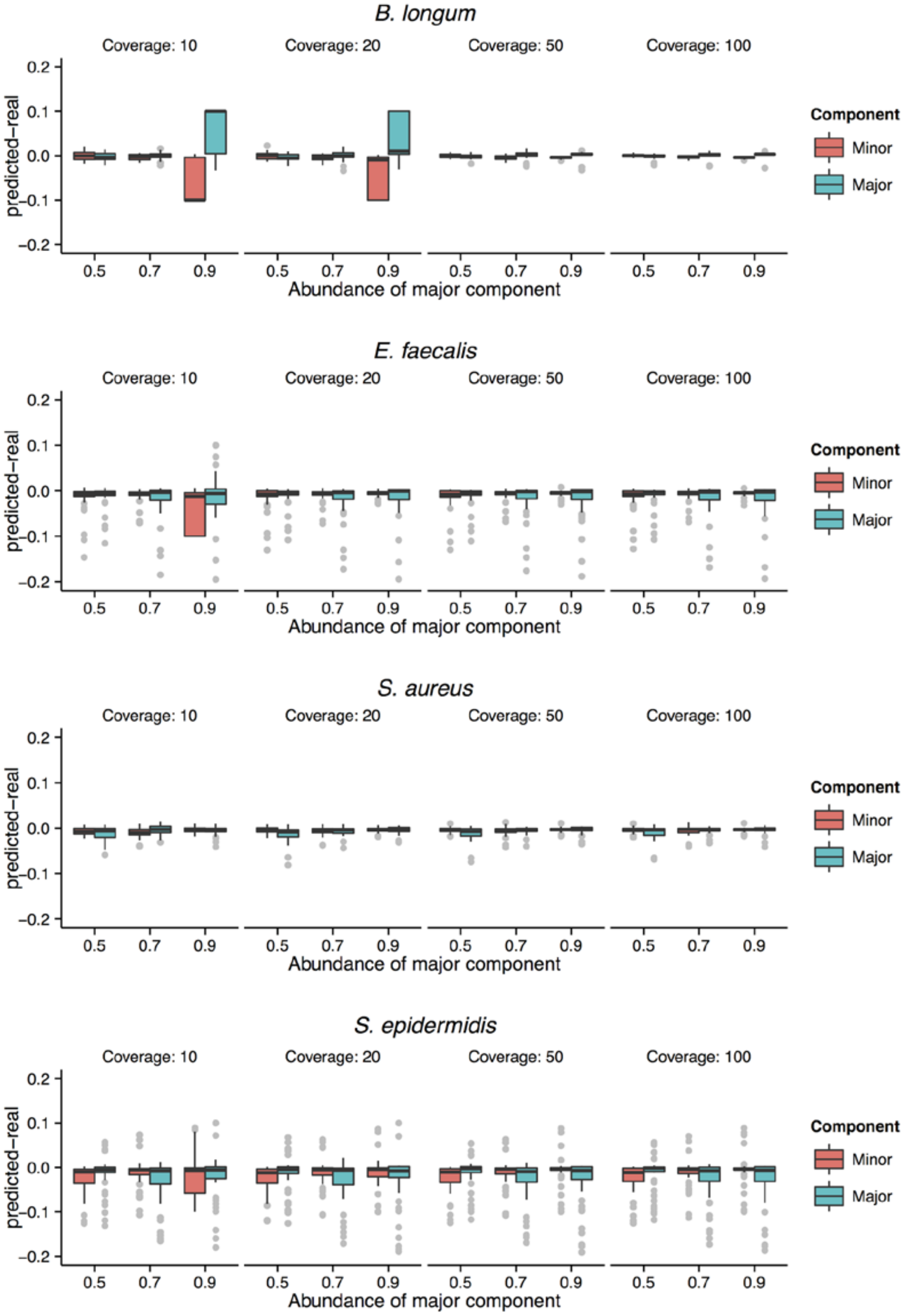

Supplementary Fig. 1. Difference between the predicted and real relative abundances for the *syntheticII dataset* plotted as a function of the abundance of the major (dominant) component. Boxes indicate the 25% and 75% percentiles while whiskers extend to the highest (lowest) value that is within 1.5 times the inter-quartile range. Outliers are shown as grey dots.

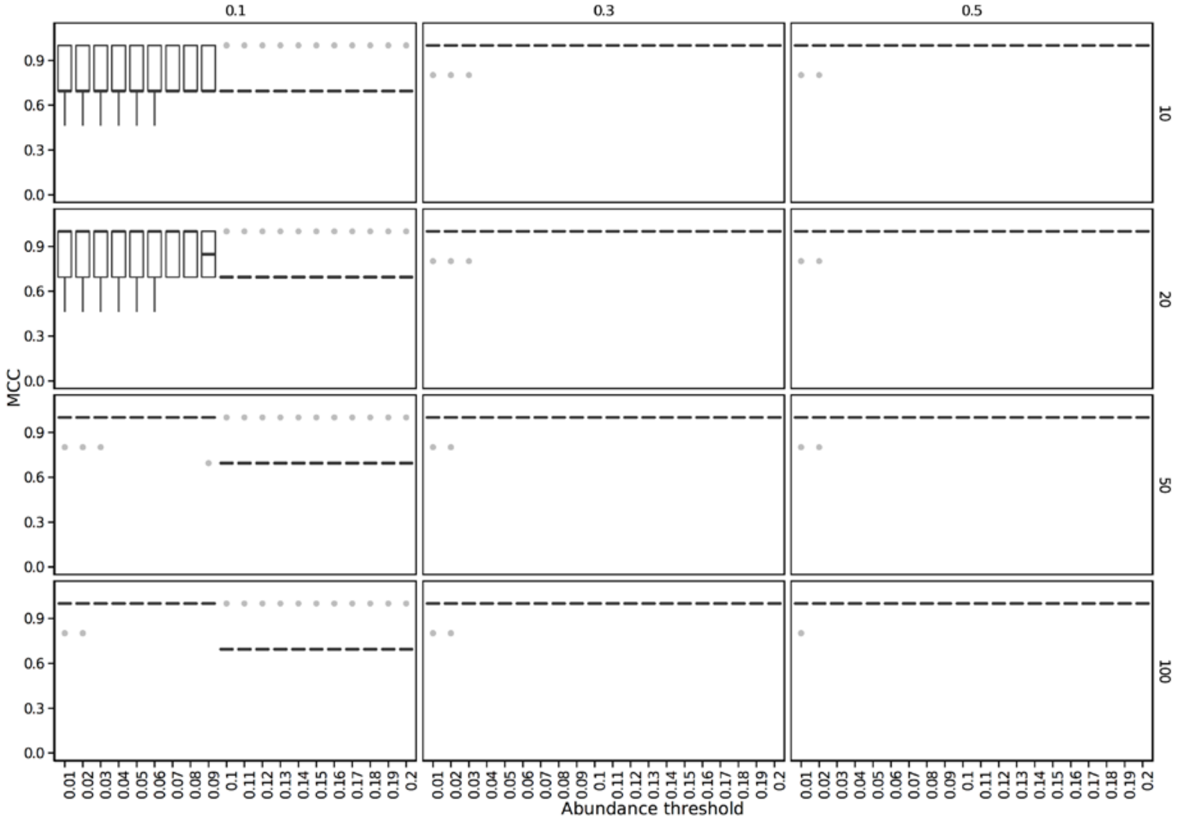

Supplementary Fig. 2. *syteticII* dataset - *B. longum*. Matthew Correlation Coefficient of the strains predicted by StrainEst for the 12 different samples with relative abundances 90%-10% (left column), 70%-30% (center column) and 50%-50% (right column) and coverage 10X (top row), 20X (second top row), 50X (third row), and 100X (bottom row). Strains are considered predicted positive if their predicted relative abundance exceeds a given threshold. The plotted data are for values of the threshold between 0.01 and 0.2. Boxes indicate the 25% and 75% percentiles while whiskers extend to the highest (lowest) value that is within 1.5 times the inter-quartile range. Outliers are shown as grey dots.

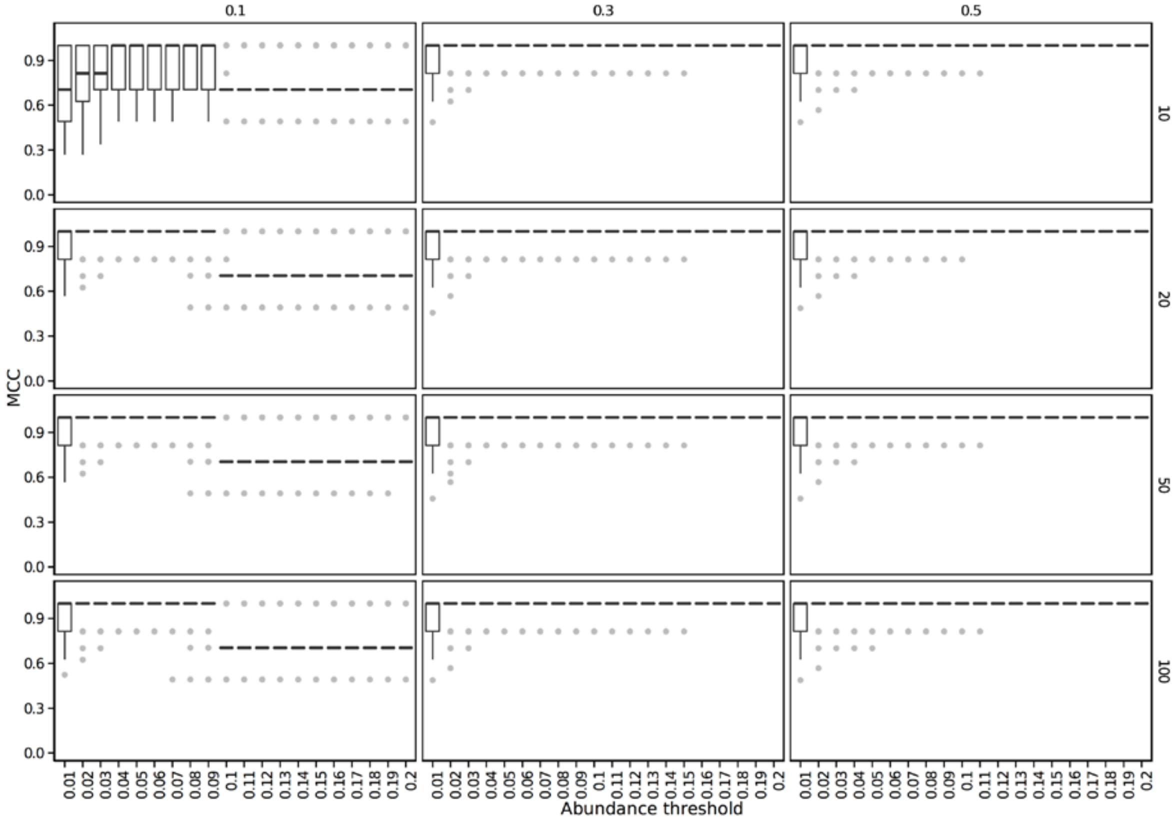

Supplementary Fig. 3. *syntheticII* dataset - E. faecalis. Same as Supplementary Fig. 2.

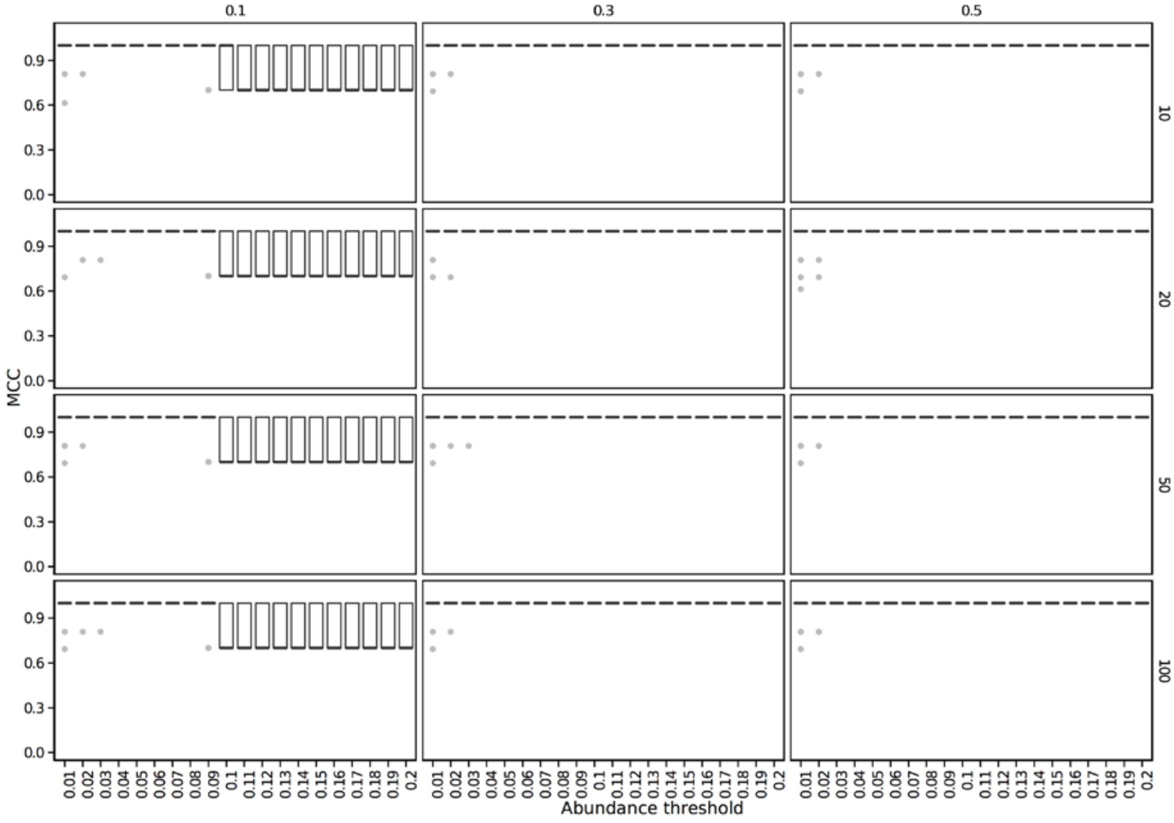

Supplementary Fig. 4. *syntheticII* dataset - S aureus. Same as Supplementary Fig. 2.

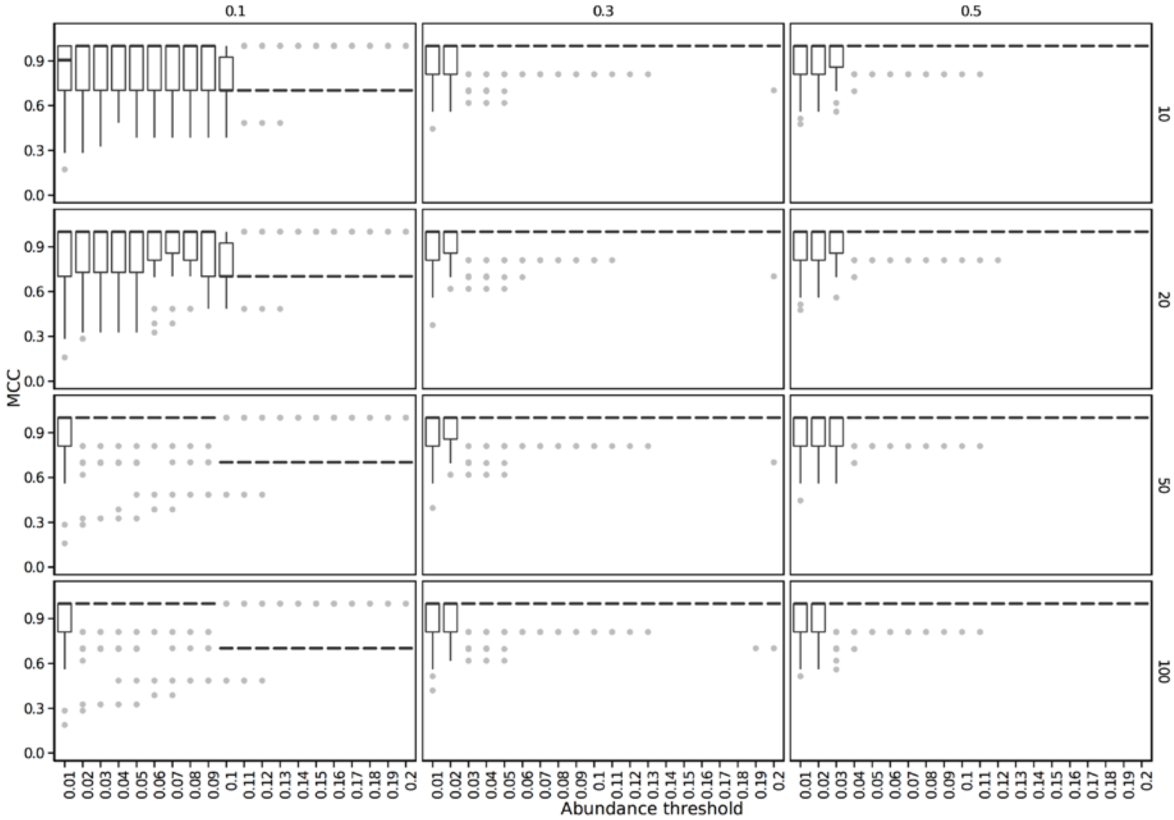

Supplementary Fig. 5. *syntheticII* dataset - S epidermidis. Same as Supplementary Fig. 2.

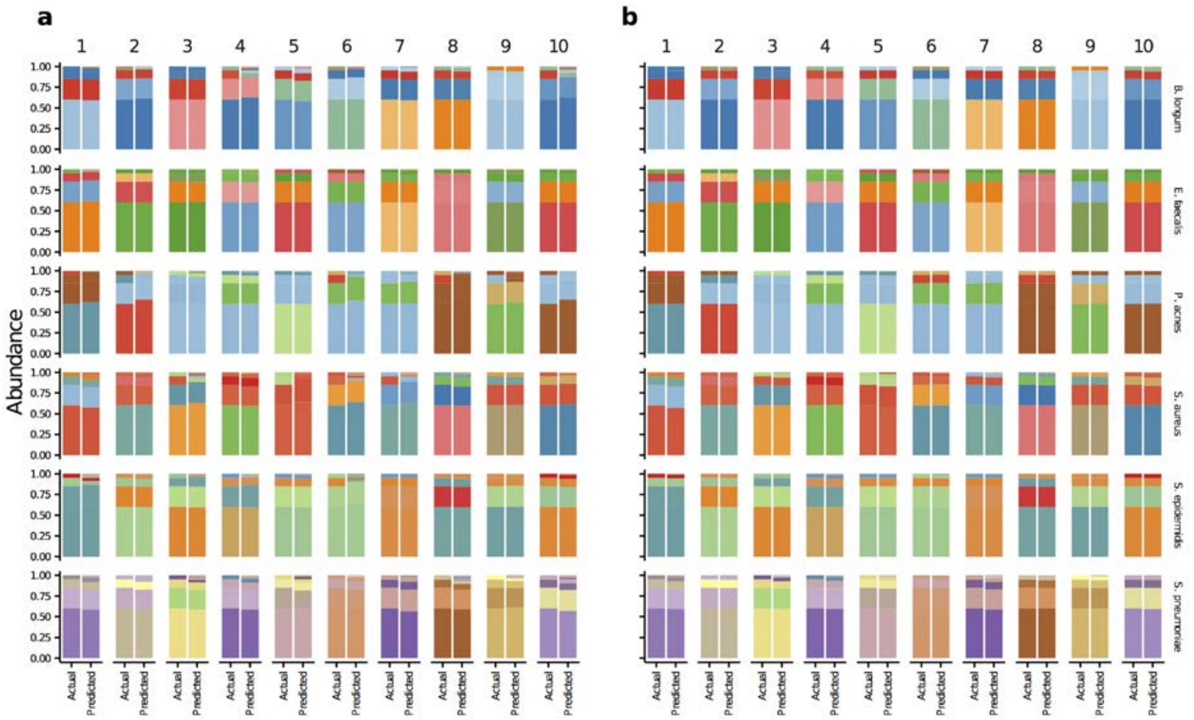

Supplementary Fig. 6. *syntheticIV* dataset. Comparison between actual and predicted relative abundances for *B. longum*, *E. faecalis*, *P. acnes*, *S. aureus*, *S. epidermidis*, and *S. pneumoniae*. For each species, we simulated 10 synthetic datasets at coverage 10X (**a**) and 100X (**b**) generating reads from four strains mixed at variable relative abundances (60-25-10-5%). Colors indicate different strains.

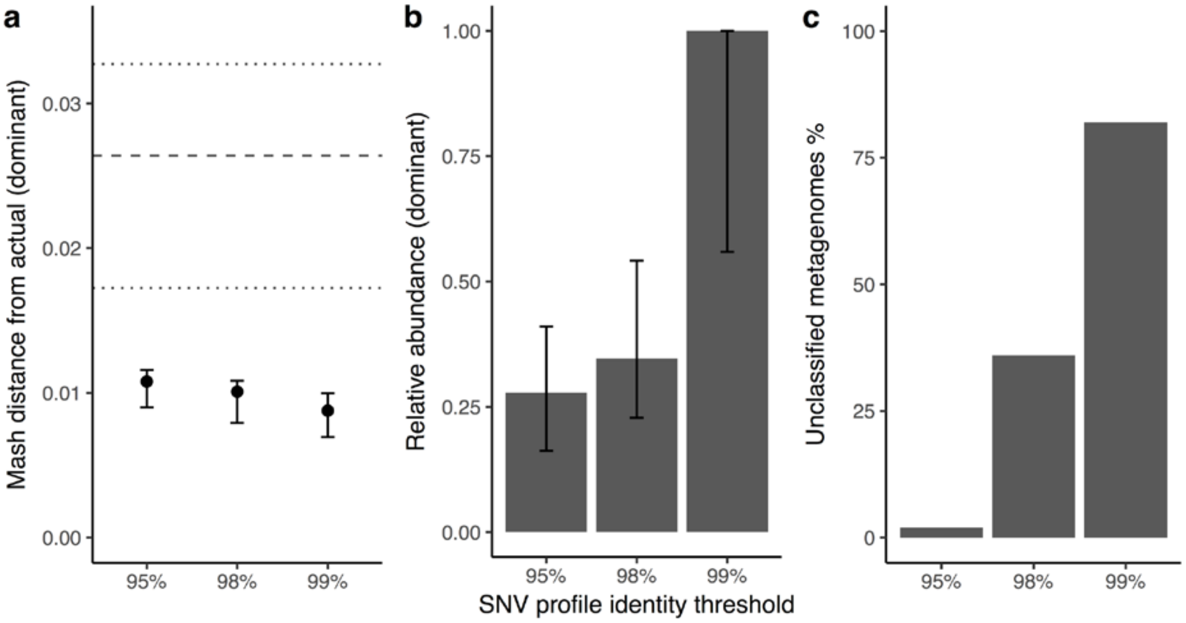

Supplementary Fig. 7. *LOOEcoli* dataset. Performances of StrainEst in the analysis of a metagenomic samples containing one strain that is absent from the reference database. Median mash distance between the predicted dominant *E. coli* strain and the actual (**a**), median estimated relative abundance of the dominant strain (**b**) and percentage of unclassified metagenomes (**c**) for three different SNV profile identity thresholds (parameter -d/--max-ident-thr). Error bars indicate the first and the third quartile. In all cases, StrainEst identified a dominant strain that was closely related to the actual. However, using the default value of the compatibility threshold StrainEst overestimated the sample complexity in an attempt to compensate for the missing strain. As the threshold increased, the accuracy of the prediction increased, but the number of predictable metagenomes decreased.

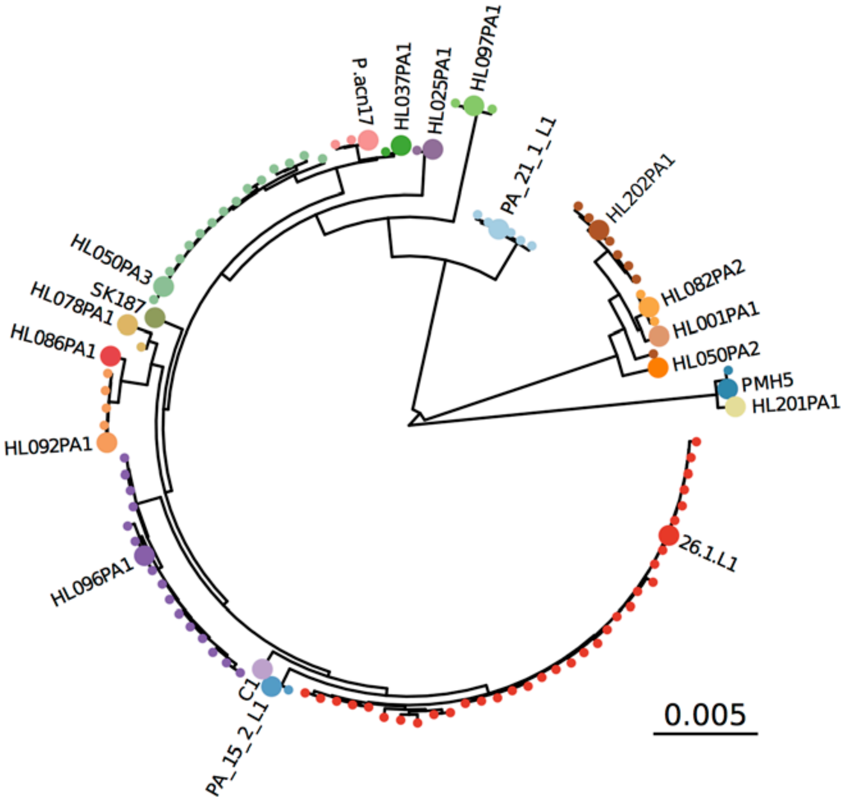

Supplementary Fig. 8. *P. acnes* Neighbor Joining tree using Mash distances. Large dots depict the representative strains after the SNV clustering steps. Colors indicates cluster membership.

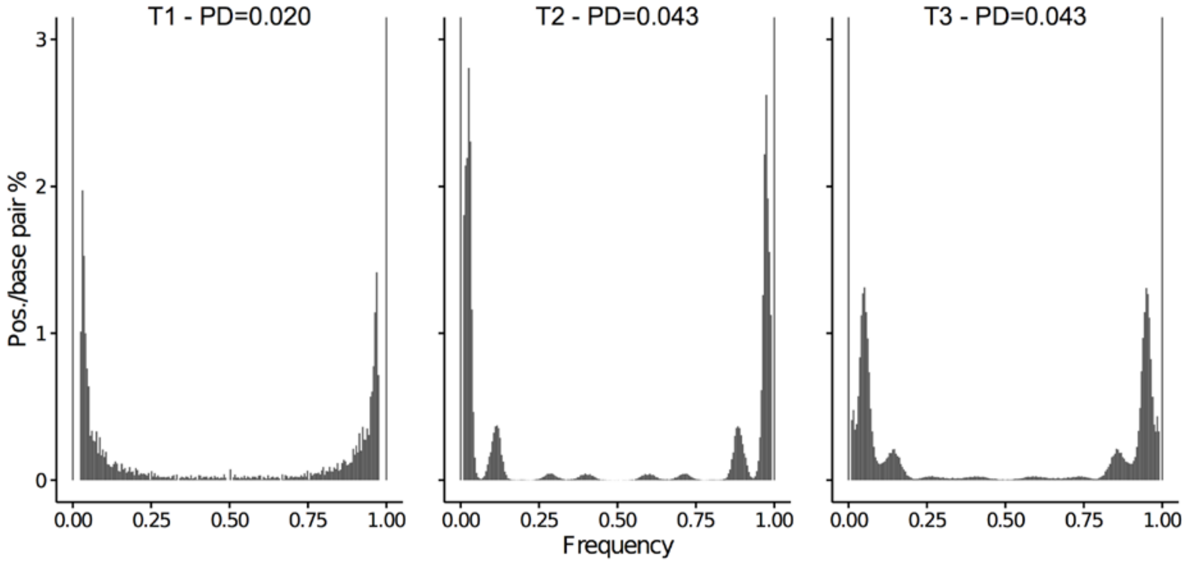

Supplementary Fig. 9. Frequency distribution of the allelic variants of P. acnes Subject HV01, Hp site for three different timepoints (T1,T2,T3). Transition from the low diversity (T1) to the high diversity (T2, T3) phenotype. In this example, the Phylogenetic Diversity increases from 0.02 (T1) to 0.043 (T2,T3). While the bi-modal frequency distribution of the allelic variants is indicative of the presence of a single strain at T1, multiple peaks appear at T2 and T3, supporting the presence of a more complex population. For clarity, the y-axis range is truncated at 3%.

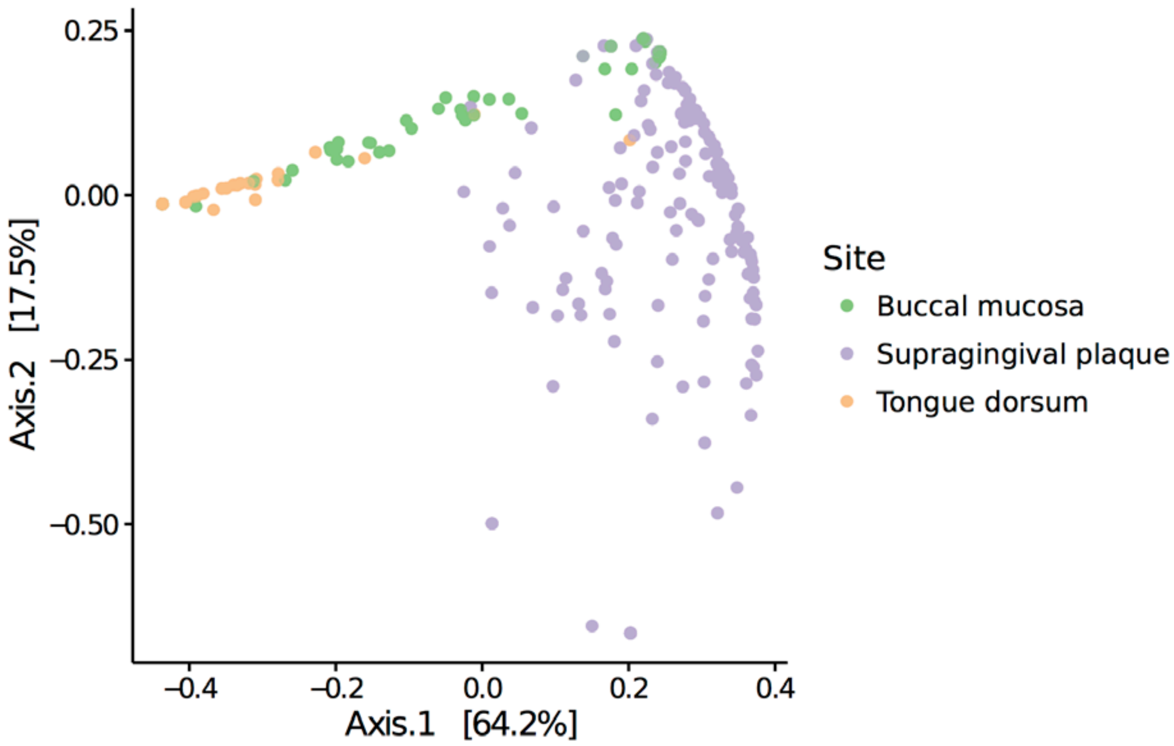

Supplementary Fig. 10. HMP oral dataset. Principal Coordinate Analysis (PCoA) using the Weighted UniFrac distances computed on the predicted relative abundances of species within the *Neisseria* genus and the phylogenetic tree estimated with the neighbor-joining method on the Mash distances. Samples with a reconstruction Pearson coefficient R<0.8 were removed from the analysis.

Supplementary Tables

	JSD		MCC	
**Species**	**Mean**	**SD**	**Mean**	**SD**
B. longum	0.0306	0.0342	0.8922	0.1602
E. coli	0.0132	0.0038	0.9786	0.0452
E. faecalis	0.0080	0.0126	0.9862	0.0436
P. acnes	0.0554	0.0448	0.6826	0.2562
S. aureus	0.0482	0.0469	0.7816	0.2587
S. epidermidis	0.0353	0.0446	0.8413	0.2286
S. pneumoniae	0.0224	0.0068	0.9492	0.0694

Supplementary Table 1. *syntheticIV* dataset (10X coverage). JSD and MCC between the actual and predicted strain composition. SD: standard deviation.

	JSD		MCC	
**Species**	**Mean**	**SD**	**Mean**	**SD**
B. longum	0.0024	0.0014	1.0000	0.0000
E. coli	0.0072	0.0044	0.9893	0.0339
E. faecalis	0.0015	0.0008	1.0000	0.0000
P. acnes	0.0024	0.0017	1.0000	0.0000
S. aureus	0.0063	0.0041	0.9655	0.0555
S. epidermidis	0.0028	0.0018	1.0000	0.0000
S. pneumoniae	0.0103	0.0045	1.0000	0.0000

Supplementary Table 2. *syntheticIV* dataset (100X coverage). Same as Supplementary Table 1.

Sample	Species	Nr. of aligned reads	Cov. SNV sites (min-max)	N. of covered SNV pos.	Representative sequence (predicted)	Strain designation	Rel. ab.
**SRR172902**	E. coli	97288	1-3	66268	GCF_000819325.1_Escherichia_coli_CVM_N38381PS_v1.0_genomic.fna	MG1655	68%
	N. meningitidis	128653	1-6	21770	GCF_000327945.fna	MC58	69%
	P. acnes	255772	2-9	100161	GCF_001469595.1_ASM146959v1_genomic.fna	DSM16379/KPA171202	100%
	S. aureus	188498	2-8	74392	GCF_000153665.1_ASM15366v1_genomic.fna	USA300_TCH959	100%
	S. epidermidis	252772	3-10	92013	GCF_000007645.1_ASM764v1_genomic.fna	ATCC 12228	98%
**SRR172903**	E. coli	558192	3-10	88130	GCF_000819325.1_Escherichia_coli_CVM_N38381PS_v1.0_genomic.fna	MG1655	68%
	N. meningitidis	12745	1-2	4428	Not converged	NA	NA
	P. acnes	19612	1-2	29304	GCF_001469595.1_ASM146959v1_genomic.fna	DSM16379/KPA171202	100%
	S. aureus	1490833	25-46	70742	GCF_000153665.1_ASM15366v1_genomic.fna	USA300_TCH959	99%
	S. epidermidis	1227894	23-39	87969	GCF_000007645.1_ASM764v1_genomic.fna	ATCC 12228	98%

Supplementary Table 3. Analysis of two Mock communities from the HMP project. For the two samples SRR172902 (even composition) and SRR172903 (staggered composition) we show the number of reads that align to the references, the coverage of the SNV positions (range, min-max), the number of covered SNV positions, the predicted dominant representative sequence, its strain designation and predicted relative abundance. Strain designation is determined by comparing the strain designation of the sequences included in the cluster represented by the sequence identified by StrainEst. With the exception of *S. aureus* and *S. epidermidis* in sample SRR172903, the coverage for all the species was always very low, never exceeding 10.

	Version	Strain-level relative abundance profiling (reference-based)	Strain-level relative abundance profiling (denovo)	Dominant strain detection	Pangenome profiling	SNV profiling
StrainEst	1.2	YES	NO	YES	NO	YES
PanPhlAn	1.2.0.6	NO	NO	YES	YES	NO
MIDAS	1.2.2	NO	NO	NO	YES	YES
ConStrains	2016-04-20	NO	YES	NO	NO	YES
PathoScope	2.0.6	YES	NO	YES	NO	NO
Sigma	1.0.1	YES	NO	YES	NO	YES

Supplementary Table 4. Analysis provided by StrainEst, PanPhlAn, MIDAS, ConStrains, PathoScope and Sigma. Both MIDAS and PanPhlAn provide a profile of the species pangenome present in metagenomic samples. ConStrains provides a denovo strain-level relative abundance profiling while StrainEst, PathoScope and Sigma perform a reference-based profiling.

Filename	Description
GCF_000083565.fna	Neisseria meningitidis alpha14 (b-proteobacteria);alpha14
GCF_000386625.fna	Neisseria meningitidis NM3144 (b-proteobacteria);NM3144
GCF_000448005.fna	Neisseria meningitidis 96037 (b-proteobacteria);96037
GCF_000293405.fna	Neisseria meningitidis 98008 (b-proteobacteria);98008
GCF_000220865.fna	Neisseria macacae ATCC 33926 (b-proteobacteria);ATCC 33926
GCF_000193755.fna	Neisseria sicca DS1 (b-proteobacteria);DS1
GCF_000156835.fna	Neisseria gonorrhoeae FA19 (b-proteobacteria);FA19
GCF_000327805.fna	Neisseria meningitidis 63049 (b-proteobacteria);63049
GCF_000193795.fna	Neisseria lactamica NS19 (b-proteobacteria);NS19
GCF_000193735.fna	Neisseria sicca 4320 (b-proteobacteria);4320
GCF_000191505.fna	Neisseria meningitidis M04-240196 (b-proteobacteria);M04-240196
GCF_000387145.fna	Neisseria meningitidis 2003051 (b-proteobacteria);2003051
GCF_000293465.fna	Neisseria meningitidis NM2657 (b-proteobacteria);NM2657
GCF_000328005.fna	Neisseria meningitidis 98080 (b-proteobacteria);98080
GCF_000328145.fna	Neisseria meningitidis NM126 (b-proteobacteria);NM126
GCF_000176735.fna	Neisseria polysaccharea ATCC 43768 (b-proteobacteria);ATCC 43768
GCF_000327785.fna	Neisseria meningitidis 65014 (b-proteobacteria);65014
GCF_000191325.fna	Neisseria meningitidis 961-5945 (b-proteobacteria);961-5945
GCF_000293385.fna	Neisseria meningitidis NM576 (b-proteobacteria);NM576
GCF_000327885.fna	Neisseria meningitidis NM174 (b-proteobacteria);NM174
GCF_000386805.fna	Neisseria meningitidis 2002020 (b-proteobacteria);2002020
GCF_000386965.fna	Neisseria meningitidis 2004032 (b-proteobacteria);2004032
GCF_000328105.fna	Neisseria meningitidis 2004090 (b-proteobacteria);2004090
GCF_000386265.fna	Neisseria meningitidis 63023 (b-proteobacteria);63023
GCF_000156975.fna	Neisseria gonorrhoeae SK-93-1035 (b-proteobacteria);SK-93-1035
GCF_000448185.fna	Neisseria meningitidis NM518 (b-proteobacteria);NM518
GCF_000327945.fna	Neisseria meningitidis M13255 (b-proteobacteria);M13255
GCF_000327865.fna	Neisseria meningitidis 9506 (b-proteobacteria);9506
GCF_000260655.fna	Neisseria sicca VK64 (b-proteobacteria);VK64
GCF_000293265.fna	Neisseria meningitidis 93003 (b-proteobacteria);93003
GCF_000191465.fna	Neisseria meningitidis M01-240149 (b-proteobacteria);M01-240149
GCF_000386945.fna	Neisseria meningitidis 2001001 (b-proteobacteria);2001001
GCF_000173995.fna	Neisseria lactamica ATCC 23970 (b-proteobacteria);ATCC 23970
GCF_000090875.fna	Neisseria sp. oral taxon 014 str. F0314 (b-proteobacteria);F0314
GCF_000174655.fna	Neisseria sicca ATCC 29256 (b-proteobacteria);ATCC 29256
GCF_000386765.fna	Neisseria meningitidis 73704 (b-proteobacteria);73704
GCF_000173955.fna	Neisseria subflava NJ9703 (b-proteobacteria);NJ9703
GCF_000293245.fna	Neisseria meningitidis 93004 (b-proteobacteria);93004
GCF_000191485.fna	Neisseria meningitidis M01-240355 (b-proteobacteria);M01-240355
GCF_000191265.fna	Neisseria meningitidis M0579 (b-proteobacteria);M0579
GCF_000186165.fna	Neisseria mucosa C102 (b-proteobacteria);C102
GCF_000191245.fna	Neisseria meningitidis M13399 (b-proteobacteria);M13399
GCF_000196295.fna	Neisseria lactamica 020-06 (b-proteobacteria);020-06

GCF_000146655.fna	Neisseria meningitidis ATCC 13091 (b-proteobacteria);ATCC 13091
GCF_000173875.fna	Neisseria mucosa ATCC 25996 (b-proteobacteria);ATCC 25996
GCF_000448165.fna	Neisseria meningitidis NM0552 (b-proteobacteria);NM0552
GCF_000173935.fna	Neisseria flavescens NRL30031/H210 (b-proteobacteria);NRL30031/H210
GCF_000176755.fna	Neisseria elongata subsp. glycolytica ATCC 29315 (b-proteobacteria);ATCC 29315
GCF_000328045.fna	Neisseria meningitidis 77221 (b-proteobacteria);77221
GCF_000006845.fna	Neisseria gonorrhoeae FA 1090 (b-proteobacteria);FA 1090
GCF_000173895.fna	Neisseria cinerea ATCC 14685 (b-proteobacteria);ATCC 14685
GCF_000293285.fna	Neisseria meningitidis NM255 (b-proteobacteria);NM255
GCF_000448085.fna	Neisseria meningitidis NM045 (b-proteobacteria);NM045
GCF_000014105.fna	Neisseria meningitidis 053442 (b-proteobacteria);053442
GCF_000386685.fna	Neisseria meningitidis NM51 (b-proteobacteria);NM51
GCF_000293625.fna	Neisseria meningitidis NM2795 (b-proteobacteria);NM2795
GCF_000293665.fna	Neisseria meningitidis NM3001 (b-proteobacteria);NM3001
GCF_000156875.fna	Neisseria gonorrhoeae PID18 (b-proteobacteria);PID18
GCF_000227275.fna	Neisseria sp. GT4A_CT1 (b-proteobacteria);GT4A_CT1
GCF_000448225.fna	Neisseria meningitidis NM3230 (b-proteobacteria);NM3230
GCF_000175275.fna	Neisseria flavescens SK114 (b-proteobacteria);SK114
GCF_000194925.fna	Neisseria bacilliformis ATCC BAA-1200 (b-proteobacteria);ATCC BAA-1200
GCF_000367485.fna	Neisseria meningitidis NMB (b-proteobacteria);NMB
GCF_000386745.fna	Neisseria meningitidis 73696 (b-proteobacteria);73696
GCF_000191425.fna	Neisseria meningitidis G2136 (b-proteobacteria);G2136
GCF_000293445.fna	Neisseria meningitidis 92045 (b-proteobacteria);92045
GCF_000191205.fna	Neisseria meningitidis OX99.30304 (b-proteobacteria);OX99.30304
GCF_000240545.fna	Neisseria meningitidis Nm8187 (b-proteobacteria);Nm8187
GCF_000156775.fna	Neisseria gonorrhoeae 35/02 (b-proteobacteria);35/02
GCF_000387105.fna	Neisseria meningitidis 2005172 (b-proteobacteria);2005172
GCF_000448065.fna	Neisseria meningitidis NM3139 (b-proteobacteria);NM3139
GCF_000026965.fna	Neisseria meningitidis 8013 (b-proteobacteria);8013
GCF_000386785.fna	Neisseria meningitidis 81858 (b-proteobacteria);81858
GCF_000413215.fna	Neisseria meningitidis NM134 (b-proteobacteria);NM134
GCF_000191345.fna	Neisseria meningitidis M01-240013 (b-proteobacteria);M01-240013
GCF_000293425.fna	Neisseria meningitidis 80179 (b-proteobacteria);80179
GCF_000327745.fna	Neisseria meningitidis 69096 (b-proteobacteria);69096
GCF_000318235.fna	Neisseria sp. oral taxon 020 str. F0370 (b-proteobacteria);F0370
GCF_000293645.fna	Neisseria meningitidis NM3081 (b-proteobacteria);NM3081

Supplementary Table 5. 79 Neisseriae genomes used as reference in the analysis of the HMP oral dataset.

**Species**	**# of downloaded genomes**	**Representative genomes selection**		**Reference SNV matrix**		
		**Mash dist. thr.**	**# of repr. genomes**	**Species repr.**	**# of ref. genomes**	**# of SNV**
B. longum	47	0.001	30	NCC2705	29	99406
E. faecalis	416	0.001	264	V583	117	109312
P. acnes	110	0.001	25	KPA171202	20	115521
S. aureus	5413	0.001	761	NCTC 8325	52	86365
S. epidermidis	278	0.001	146	ATCC 12228	67	107194
Escherichia coli	3041	0.006	544	K-12 substr. MG1655	278	104248
Neisseriae	212	0.004	85	Neisseria meningitidis MC58	79	25393

Supplementary Table 6. Selection of the representative genomes for SNV profiling. The Mash distance threshold from the species representative is the threshold used for the preliminary clustering from the pairwise Mash distance matrix (see Fig. 1a, main text). This clustering yields a set of representative genomes that are aligned against the species representative using NUCmer to identify the core genome and the set of SNVs. Reference SNV profiles are finally clustered obtaining the SNV matrix used in the modeling step (see Fig. 1b and 1c, main text).

**Species**	**Q_1**	**Q_2 (median)**	**Q_3**
B. longum	80.9375	82.3550	82.9575
E. faecalis	87.0450	89.0950	89.9650
P. acnes	94.4075	96.1950	96.5050
S. aureus	87.3125	88.4750	89.8750
S. epidermidis	89.3575	91.4450	92.5325
S. pneumoniae	84.1775	85.7600	89.0325
Escherichia coli	79.1075	81.1150	81.7625

Supplementary Table 7. *syntheticIV* dataset (100X): alignment rates (*i.e.*percentage of aligned reads) against a database including 10 representative sequences.Q_1: first quartile, Q_2: median, Q_3: third quartile. For all species, the choice of 10 reference sequences guarantees that at least 80% of the reads are aligned. The number of reference sequences can be increased to improve sensitivity.

**Species**	**# of ref. genomes in the SNV matrix**	**# of SNV**	**Coverage**	**Running time [sec]**	**Maximum memory occupied [MB]**
B. longum	29	99406	10	781	129
			20	1081	130
			50	841	129
			100	1141	129
S. aureus	52	86365	10	721	154
			20	781	154
			50	901	235
			100	961	231
S. epidermidis	67	107194	10	1201	437
			20	901	447
			50	1141	438
			100	1502	438
E. faecalis	117	109312	10	1081	406
			20	1141	591
			50	1261	446
			100	1382	453

Supplementary Table 8. Execution time and maximum required memory by the modeling step (command strainest est) for four *syntheticII* samples. StrainEst was run on a desktop machine with an Intel® Core™ i7-3770, 4 cores and 16 GB of RAM.

Supplementary Methods

Comparison to existing tools

To compare the performances of StrainEst to existing tools, we run ConStrains, PanPhlAn, PathoScope, Sigma, and Bowtie 2 on the 50 independent samples of the *syntheticEcoli* dataset.

ConStrains

ConStrains (version 2016-04-20) was run using the default parameters and MetaPhlAn2 version 2.6.0:

```
ConStrains.py -m metaphlan2.py -c sample.conf -o output
```

PanPhlAn

We downloaded the *E. coli* pangenome database from `https://bitbucket.org/CibioCM/panphlan/wiki/Pangenome%20databases`. Metagenomic samples were mapped against the *E. coli* pangenome using PanPhlAn version 1.2.0.6:

```
cat read1.fastq read2.fastq > read.fastq
panphlan_map.py -c ecoli16 --i_bowtie2_indexes \
    $BOWTIE2_INDEXES -i read.fastq -o map_results/output.csv
```

For each *E. coli* dataset (2, 3 and 4 strains) the mapping results were merged and processed for getting the final gene-family presence/absence profile matrix:

```
panphlan_profile.py -c ecoli16 -i map_results \
    --i_bowtie2_indexes $BOWTIE2_INDEXES --o_dna \
    result_gene_presence_absence.csv
```

The dominant strain was determined as the strain with the minimum Jaccard distance between gene family profiles of the reference strains and the metagenome.

PathoScope

We downloaded the nt_02_04_2016_ti.fa reference database from `ftp://pathoscope.bumc.bu.edu/data/` and created a *E. coli* specific PathoScope (version 2.0.6) database with the command:

```
python pathoscope2.py LIB -genomeFile nt_02_04_2016_ti.fa \
    -taxonIds 562 --subTax -outPrefix E_coli
```

for each sample dataset we then run the mapping step:

```
python pathoscope2.py MAP -1 read1.fastq -2 read2.fastq \
    -targetRefFiles E_coli_ti.fa -outDir results_sample \
    -outAlign sample.bam -expTag sample
```

and then the prediction step using the informative prior[12]:

```
pathoscope2.py ID -alignFile sample.bam -fileType sam \
    -outDir results_sample -expTag sample -thetaPrior 10**88
```

Sigma

Sigma (version 1.0.1) was run using the default configuration file. The Sigma reference genome database was constructed from the complete set of 287 reference genomes used by StrainEst:

```
sigma-index-genomes -c sigma_config.cfg
```

After that, metagenomic reads were aligned against the reference database and the probabilistic model was built and solved:

```
sigma-align-reads -c sigma_config.cfg -w output_dir
sigma-build-model -c sigma_config.cfg -w output_dir
sigma-solve-model -c sigma_config.cfg -w output_dir -i \
    output_dir/sigma_out.qmatrix.txt
```

Bowtie2

The Bowtie2 (version 2.2.9) index was built from the complete set of 287 *E. coli* reference genomes used by StrainEst. For each metagenome, a Bowtie2 alignment against the references was performed. Reads with a mapping quality score (MAPQ) <10 were removed and the read counts for each reference sequence were finally extracted:

```
bowtie2 --no-unal -x ecoli -1 read1.fasta -2 read2.fasta \
    -S bowtie2_out_tmp.sam
samtools view -b bowtie2_out_tmp.sam > bowtie2_out_tmp.bam
samtools view -b -q 10 bowtie2_out_tmp.bam > bowtie2_out.bam
samtools sort bowtie2_out.bam -o bowtie2_out_sorted.bam
samtools index bowtie2_out_sorted.bam
samtools idxstats bowtie2_out_sorted.bam > counts.txt
```

For each metagenomic sample, the dominant strain and the secondary components were determined naively ranking the 278 reference genomes according the number of aligned reads.
