# Supplementary material for: Strain profiling and epidemiology of bacterial species from metagenomic sequencing
Source: Nat Commun. 2017 Dec 22;8:2260. doi: 10.1038/s41467-017-02209-5 (PMC5741664; doi:10.1038/s41467-017-02209-5)
Supplement: Supplementary file 2 — Description of Additional Supplementary File [file 41467_2017_2209_MOESM2_ESM.pdf]

Description of Additional Supplementary File

File Name: Supplementary Data 1
Description: Prediction accuracy in terms of Mash distances and relative abundance estimation between the actual and the predicted strains of Bowtie2, PanPhlAn, PathoScope and StrainEst on the *syntheticEcoli* dataset.
